# Environmentally benign fabrication of SnO_2_-CNT nanohybrids and their multifunctional efficiency as an adsorbent, catalyst and antimicrobial agent for water decontamination

**DOI:** 10.1038/s41598-019-49181-2

**Published:** 2019-09-10

**Authors:** Md. Ahmaruzzaman, Dipyaman Mohanta, Abhijit Nath

**Affiliations:** 1 Department of Chemistry, National Institute of Technology, Silchar, Assam 788010 India; 2 Department of Chemistry, G.C. College, Silchar, Assam 788004 India

**Keywords:** Pollution remediation, Synthesis and processing

## Abstract

Herein, we described a biogenic, additive fee, eco-friendly synthesized SnO_2_-CNT nanohybrid as an efficient, re-collectable and reusable material for onsite water remediation. We demonstrated that the SnO_2_-CNTs can provide a one stop solution for water remediation as it effectively accomplished the major treatment tasks like adsorption, catalytic transformation/degradation and disinfection. The structural, morphological, surface chemical compositions of the nanocomposite and the adsorption, catalytic and antimicrobial properties were investigated using common characterization and instrumental techniques. The results revealed the brilliant efficiency of SnO_2_-CNT nanoadsorbent towards As (III) and a maximum Langmuir adsorption capacity of 106.95 mg/g was observed at high arsenite concentration (C_0_ = 1 mg/L). The nanoadsorbent was also found to be equally efficient in low arsenite concentration ranges (C_0_ = 100 μg/L) as it could bring down the arsenic concentration below maximum permissible limit. Moreover, using model pollutants like p-nitrophenol, Alizarin red S, Metronidazole, bacterial strains (*Bacillus subtilis*, *Escherichia coli*, *Streptococcus pneumonia etc*.), *and* fungal strains (*Aspergillus niger* and *Candida albicans)*, the multifunctional capability of SnO_2_-CNT towards water decontamination has been established. Our results suggested the promising potential of hierarchical nano-heterojunctions for engineering efficient water treatment processes.

## Introduction

The well-being of mankind is being alarmingly threatened by the increasing environmental crisis and the cardinal being the scarcity of clean drinking water. Rapid urbanization and illimitable human interventions have led to contamination of natural water sources leading to ecological disarray and jeopardizing both aquatic and humanoid biomes^1^. Activated carbons from various natural feed stocks have been extensively utilized for water decontamination because of their low cost, easy synthesis, highly porous nature and good adsorption capacity^2^. However, the microscopic pores of activated carbon often get blocked after several runs of adsorption-desorption process, thereby, decreasing adsorption efficiency. Carbon nanotubes (CNTs) have immense potential to substitute activated carbons and have been believed to revolutionize water treatment technologies in near future. The open structure of CNTs offers high surface area, faster kinetics and higher adsorption capacity due to the easy and undisrupted access of the contaminants to the reactive sites^3^. Moreover, introduction of surface functionalities such as -OH, -COOH *etc*., and hybridization with various metal oxide nanoparticles like Fe_2_O_3_, ZrO_2_, CeO_2_, MnO_2_ *etc*., can dramatically improve the adsorption behavior^4–7^.

However, development of cost effective and environment friendly fabrication methodologies is one of the biggest challenges to the widespread applicability of CNT based nanoadsorbents. Chemical vapor deposition (CVD) is probably among the most effective methods available for the low cost and large scale synthesis of CNTs. Recently, various natural carbon feedstocks such as palm oil, turpentine oil, clarified butter, chicken fat *etc*. have been successfully utilized for the synthesis of CNTs by CVD process^8–11^. Moreover, various bio resources like plants extract, bacterial proteins, biomolecules *etc*. have been utilized to control the size and aggregation of nanoparticles. This enabled the green and mass production of CNT-metal oxide composite feasible^12–14^.

Herein, SnO_2_-CNTs were fabricated employing a biosynthetic procedure, utilizing, sunflower oil as a carbon source and *Coccinia grandis* extracts as complexing and stabilizing agent. The as synthesized SnO_2_-CNT nanocomposites were exploited to perform multidimensional functions in water treatment including adsorption of arsenic, disinfecting pathogenic microorganisms and catalytic transformation/degradation of organic pollutants.

Arsenic is one of the most toxic contaminants found in natural water and is widespread in many regions around the world. In India, north-eastern states and some parts of west Bengal are at high risk with ground water contamination^15^. According to World Health Organization (WHO) guidelines, the arsenic content in drinking water should be less than 10 μg/L. However, millions of peoples worldwide drink water with a higher arsenic concentration, thereby suffering from long term health issues like cancers of the skin, liver, lung, and bladder, cardiovascular diseases, neurotoxicity and *etc*.^16^. As (III) is the dominant species in ground water and because of its nonionic existence (H_3_AsO_3_), it has little affinity towards the most of the adsorbents. Therefore, pre-treatment oxidation of As (III) to As (V) thus enhances arsenic removal efficacy by many water treatment processes^17^. The photocatalytic oxidation of As (III) to As (V) over semiconducting nanoparticles offers an environmentally benign method for such preoxidation. TiO_2_ assisted photocatalytic oxidation of As (III) has been widely reported^18–20^. Various hybrid materials like TiO_2_ coated CNTs, CeO_2_-CNTs, Fe_2_O_3_-CNTs *etc*. have also been utilized for rapid and effective arsenic sorption^21–23^. However, reports on oxidative adsorption of As (III) over composite nanomaterials are very scant. Therefore, it is desirable to develop low cost photocatalytic adsorbent capable of oxidizing As (III) to As (V) under UV irradiation followed by simultaneous adsorption of arsenic.

Besides heavy metals, various organic contaminants like dyes, pharmaceuticals, pesticides *etc*. are environmentally redundant materials and are serious threat to biosphere. Catalytic transformation and photocatalytic degradation of these hazardous contaminants to mineralized products is an efficient method of decontamination of water^24,25^. The catalytic reduction of nitroarenes is being widely used as a touchstone reaction to testify the catalytic efficiency of a catalyst. Also, 4-nitrophenols are considered as toxic, carcinogenic and teratogenic elements, cause impairment to liver, central nervous system, kidney etc^24,26^. Moreover, textile dyes like Alizarin red S and pharmaceutical compounds like Metronidazole were potentially carcinogenic and prone to mount up in aquatic biome, thereby leading to water pollution^27,28^. Therefore, complete remediation of these organic contaminants from water is indeed compulsory.

Additionally, development of multidrug-resistant microorganisms, especially the pathogenic bacteria and fungi at an alarming rate is a matter of global health concern. Hence, new substitute and safe antimicrobial agents to treat drinking water against human microbial pathogens is of pressing demand. Due to the advancement of antimicrobial nanothrapy, new bioengineered nanoparticles have emerged which showed antimicrobial activity due to degradation of cell membranes, alteration/destruction of cell walls, and nucleic acids. In that regard, Addae *et al*.^29^ and Sinha *et al*.^24^ reported destruction of cell walls of *Bacillus* species by Au/CuS and Ag-SnO_2_NPs. Recently, the broad spectrum antimicrobial activities of CNTs have also been established against *E*. *coli*, *S*. *aureus*, *Klebsiella pneumonia*, *S*. *agalactiae*, *Salmonella typhimurium*, and. *S*. *dysgalactiae*^30–32^. In addition, CNTs composites for example Graphene-CNT-Iron oxide, ZnO-MWCNTs, MWCNTs-CdS, MWCNTs-Ag_2_S^33–35^ have also been found to enhance broad spectrum antimicrobial activities may be due to synergism. The high potency of nanomaterials as antiviral, antibacterial, antifungal and antiprotozoal agents has thus revolutionized the pharmacological therapy.

Hence, in this work, we present a facile, bio-mediated, and economically viable fabrication of SnO_2_-CNT nanocomposite and their multifunctional capabilities such as oxidative adsorption of As (III), catalytic transformation of nitroarenes, photocatalytic degradation of Alizarin red S dye, Metronidazole and antimicrobial competence. The structure-property relationship of the surface decorated CNTs with SnO_2_ nanopartcles have been thoroughly investigated and are efficiently utilized in eradication of water contaminants.

## Experimental Section

### Materials and instruments

Fresh sunflower oil and *Coccinia grandis* leaves were obtained from local farmers market. Stannic chloride pentahydrate (SnCl_4_.5H_2_O) was obtained from Sigma Aldrich and used without further purification.

Fourier transform infrared (FTIR) spectrum was taken by a 3000 Hyperion Microscope with Vertex 80 FTIR System (Bruker, Ettlingen, Germany) spectrometer. Transmission electron microscopy (TEM) was carried out on JEOL, 9JSM-100CX equipment with an accelerating voltage of 60–200 kV. Energy dispersive X-ray (EDX) analysis was performed using the same instrument. Scanning electron microscopic (SEM) images were obtained on a JEOL, JSM-6360 equipment, LEO, 1430vpequipment and on Quanta 150 equipment with an accelerating voltage of 1KV-30KV. Powder X-ray diffraction (XRD) patterns were obtained using Philips X’PERT powder X-ray diffractometer with Cu-Kα radiation (λ = 1.54056 Å) with a scan speed 2°/min at room temperature. Raman Spectra were recorded on a Renishaw RM1000B LRM using a 514.5 nm (E_laser_ = 2.41 eV) Ar^+^ laser excitation source. Nitrogen adsorption-desorption measurements were performed using a Quanta Chrome Nova 1000 gas adsorption analyzer. X-ray photoelectron spectroscopy (XPS) of the material was performed by PHI 5000 Versa Prob II spectrometer. pH measurement of solutions was carried out on a pH meter (Macro Scientific Works (Regd), New Delhi). The arsenic adsorption process was monitored using graphite furnace atomic absorption spectrometer (ANALYTIKJENA AG VARIO 6). The absorbance spectra of the samples were recorded using GENESYS 10S UV–visible spectrophotometer. HRLCMS was recorded using 1290 Infinity UHPLC System, Agilent Technologies, USA. Elementar Liqui TOC analyzer was used to perform TOC analysis. Electron spin resonance (ESR) spectra were recorded using JEOL JES-FA200, Japan. The Photo Luminescence (PL) spectra were taken using Hitachi F4600 instrument.

The *in vitro* antimicrobial screening incorporated the fungal strains, viz., *Candida albicans* and *Aspergillus niger* and bacterial strains,viz., *Escherichia coli*, *Bacillus subtilis*, *Staphylococcus aureus* susp. *Aureus*, *Streptococcus pneumonia* and *Pseudomonas aeruginosa*. The stains were sub-cultured by Muller Hinton Broth (MHB) and maintain at 5 °C.

### Synthesis

#### Catalyst free synthesis of MWCNT (Multiwalled catbon nanotube)

The MWCNTs were prepared by chemical vapor deposition (CVD) method without using any external catalyst. A quartz boat loaded with sunflower oil (~10 g) was kept in a lower-temperature zone (300 °C) and an empty quartz boat was kept at a higher temperature zone (700 °C) in a horizontal quartz tube^10^. Argon gas was then purged at a rate of 6 cm^3^/min. The furnace was heated to 700 °C at a rate of 7 °C/min for 2 hours and then allowed to cool to ambient temperature whereupon the CNTs were obtained as black powder (yield ~3.0 g).

#### Synthesis of SnO_2_-MWCNT nanohybrids

**Figure 1:**
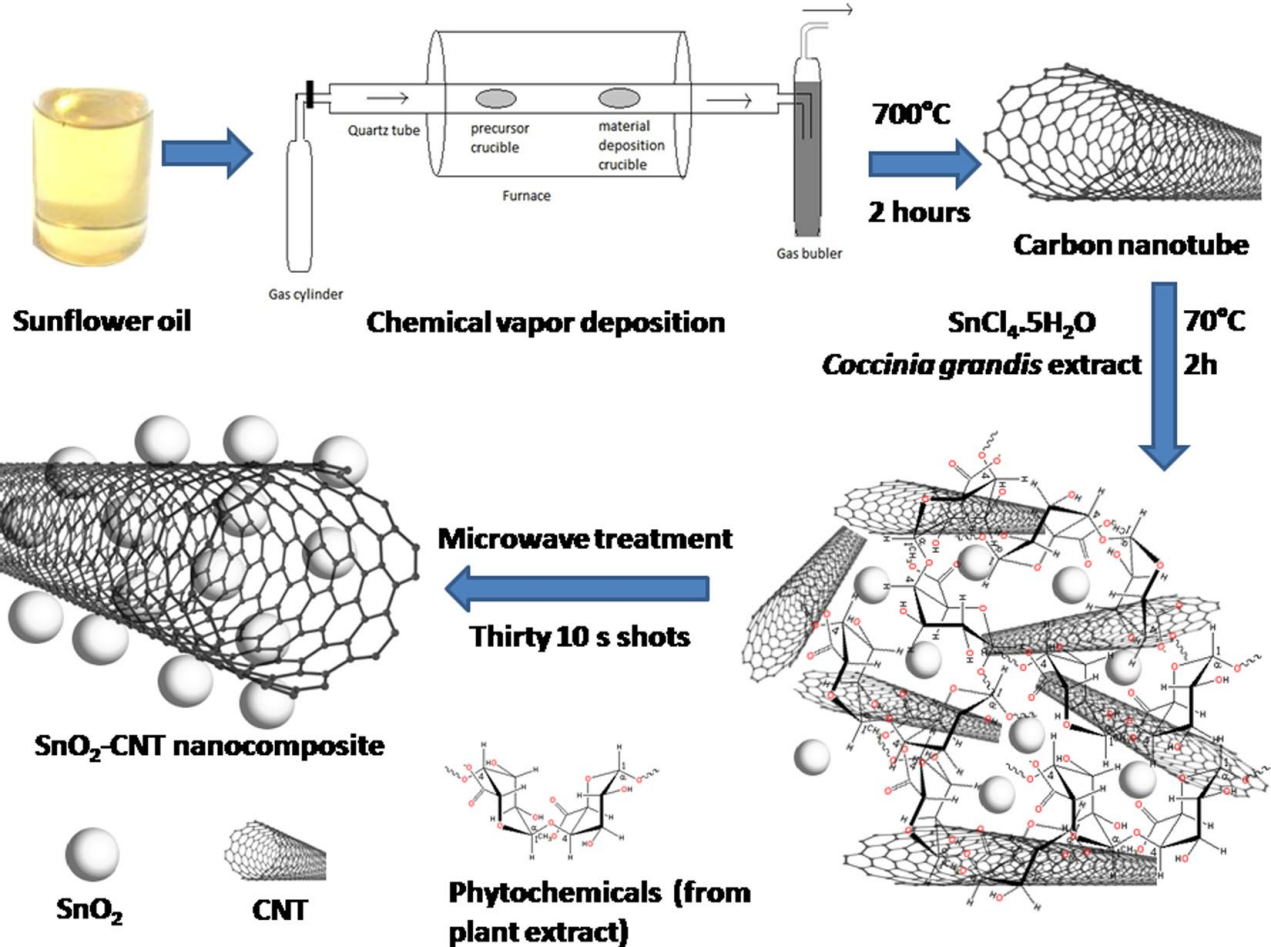
Schematic representation of the bio-mediated synthesis of SnO_2_-CNT nanocomposite.

*Coccinia grandis* leaf extract was prepared by boiling small pieces (wt. 2.5 g) of it in 100 mL of distilled water and filtering it through Whatman 40. A transparent sol was prepared by dissolving 3.0 g of SnCl_4_.5H_2_O in a minimum volume of distilled water. A calculated amount of MWCNT was added to it to get 1:1 weight ratio of the components and sonicated for about 30 minutes in order to obtain the homogeneously dispersed mixture. It was then added drop wise to the *Coccinia grandis* extract with constant stirring and maintaining a constant temperature of 70 °C for 2 h. The as obtained opal solution was then kept in a microwave oven and irradiated with thirty 10 s shots. This resulted in the precipitation of grayish mass which was centrifuged, washed several times with distilled water and air dried (yield ~4.5 g). No surfactants or stabilizing agents were used during the synthesis. The various phytochemicals present in plant extract acted as a complexing and stabilizing agent which can control the growth of the SnO_2_ nanoparticles in the reaction mixture **(**Fig. 1**)**^10,13^.

### Oxidative adsorption of As (III) on SnO_2_-CNT nanoadsorbent

All the adsorption studies were done in batch mode on a rotary shaker fitted within UV chamber. Three Philips HZ147 11W UV Lamp of wavelength 260 nm with surface irradiance of 34.72 W/m^2^ was utilized for photocatalytic oxidation of As (III). Calculated amounts sodium arsenite (NaAsO_2_) were dissolved in 1 L of distilled water to have 100 μg/L and 1 mg/L As (III) stock solutions. The arsenic adsorption was studied with 50 mL of the stock solution at pH 7.0 ± 0.1 under room temperature. The optimum amount of adsorbent for different concentrations was determined by dispersing varied amounts of adsorbent in 50 mL of As (III) solution for 300 minutes under constant mechanical stirring (50 rpm). For kinetics studies, optimum doses of adsorbent (0.1 g/L for 100 μg/L and 1.0 g/L for 1 mg/L) were dispersed in 50 mL of As (III) solutions and samples were collected in different times ranging from 0–240 min (in 15 min intervals), filtered and analyzed for arsenic using atomic adsorption spectrometer. The effect of change in concentration on adsorption behavior was examined by varying concentration in the range 40–100 μg/L and 0.4–1.0 mg/L respectively. Effects of competing ions and initial pH on arsenic removal were tested by varying interfering ions and pH respectively. The regeneration potential of the adsorbent was also investigated by five consecutive adsorption-desorption cycles. All batch experiments were performed in triplicate and the average values were reported.

### Evaluation of catalytic and photocatalytic properties of the nanocomposite

The catalytic hydrogenation of 4-nitrophenol to 4-aminophenol was used to investigate the catalytic efficiency of nanocatalysts. In a typical reaction, 1.5 mL of 3 × 10^−4^ M aqueous solution of 4-nitrophenol and 0.5 mL of 3 × 10^−2^ M NaBH_4_ solution were mixed in a quartz cuvette to have concentrations of 2.33 × 10^−4^ M and 7.5 × 10^−3^ M respectively. Then, 0.1 mg of the SnO_2_-CNT nanocatalyst was introduced into the reaction mixture and the disappearance of the yellow color was monitored using UV-Visible spectrophotometer.

The photocatalytic properties of SnO_2_-CNT were investigated by means of photodegradation of Alizarin red S and Metronidazole as water pollutants. The UV photo-reactor was consisted of three Philips HZ147 11W UV Lamp of wavelength 260 nm as a light source. The irradiance on the surface of the reaction mixture was calculated to be 34.72 W/m^2^. The experiments were performed using 10 mg/L to 80 mg/L solutions Alizarin red S and Metronidazole separately with various quantities of catalyst. The solutions were sonicated for about 60 min in dark to establish the adsorption equilibrium and then exposed with UV radiation over different time under magnetic stirring condition. The progress of the degradation process was monitored using UV-Visible spectrophotometer.

All the experiments were carried out at room temperature. The reproducibility of the results has been verified by repeating the experiments three times and an average error of ±3 was observed for reduction and degradation process.

### Evaluation of antimicrobial activity

Agar well diffusion technique was employed for antimicrobial activity of SnO_2_-CNT nanohybrids. Freshly prepared Potato dextrose agar and Muller Hinton Agar media were utilized for fungal and bacterial culture respectively. Approximately 6 mm wells were made in the solidified media and samples (50 µL) of three different molar concentrations were filled in three such wells. Azithromycin, Ciprofloxacin, Metronidazole and Cephalexin were utilized as positive control for bacteria and Nystatin and Ketoconazole for fungi. DMSO was used as negative control. The dishes were incubated for 47 h at 24 °C for fungal culture and 24 h at 36 °C for bacterial culture. After incubation, the zone of inhibitions (ZOI) was measured in millimeter (mm).

## Results and Discussion

### Role of *Coccinia grandis* leaf extract in deposition of SnO_2_ nanoparticles over CNT surface

Phytosynthesis of nanoparticles utilizing plant resources is an emerging method to reduce toxicity of nanoparticles commonly associated with conventional synthesis^36^. Plants are known to nurture a wide range of metabolites. The various phytochemicals present in plant play a crucial role in formulating and enhancing bioactivity of the nanoparticles^37^. The aqueous extracts of Coccinia *grandis* leaves are reported to have flavonoids, proteins and amino acids, glycosides, polyphenolics, carbohydrates, alkaloids *etc*.^38,39^. These phytochemicals have polar functional groups such as hydroxyl, aldehydes, ketones, esters, amides along with the carbon backbone as evident from the FT-IR spectrum of the aqueous leaf extract (Fig. S1 ESI). The chelation effect of theses functional groups may lead to the complexation or capping of the Sn^4+^ ion with the phytochemicals^37^. The nucleation phase begins with hydrolysis-condensation reactions and on microwave irradiation, the sudden blast of energy breaks the complex to form SnO_2_ nanoparticles^37,40^. The SnO_2_ nanoparticles then undergo growth and stabilization phase wherein certain phytochemicals prevent the self agglomeration to form extremely small spherical SnO_2_ nanostructures (Fig. S2 ESI). The surface functionalities of CNTs may encourage the growth and stabilization phase of SnO_2_ nanoparticles on its surface thereby leading to the formation of stable SnO_2_-CNTnanohybrid^41^. Also, a comparative account of TEM micrographs of SnO_2_-CNTnanohybrid before and after the microwave irradiation has been represented in Fig. S3 ESI. It was evident that before microwave irradiation, SnO_2_ nanoparticles were agglomerated and not evenly distributed over CNT surface. However, TEM micrograph of post microwave treatment has revealed the effective incorporation of SnO_2_ nanoparticles into the CNT. Thus, under microwave irradiation, the molecular vibrations and interfacial polarization have lead to the effective incorporation of guest into host and generation of more defect sites and heterojunctions in SnO_2_-CNT nanocomposite^42^.

### Characterization of the synthesized SnO_2_-CNT nanocomposites

**Figure 2:**
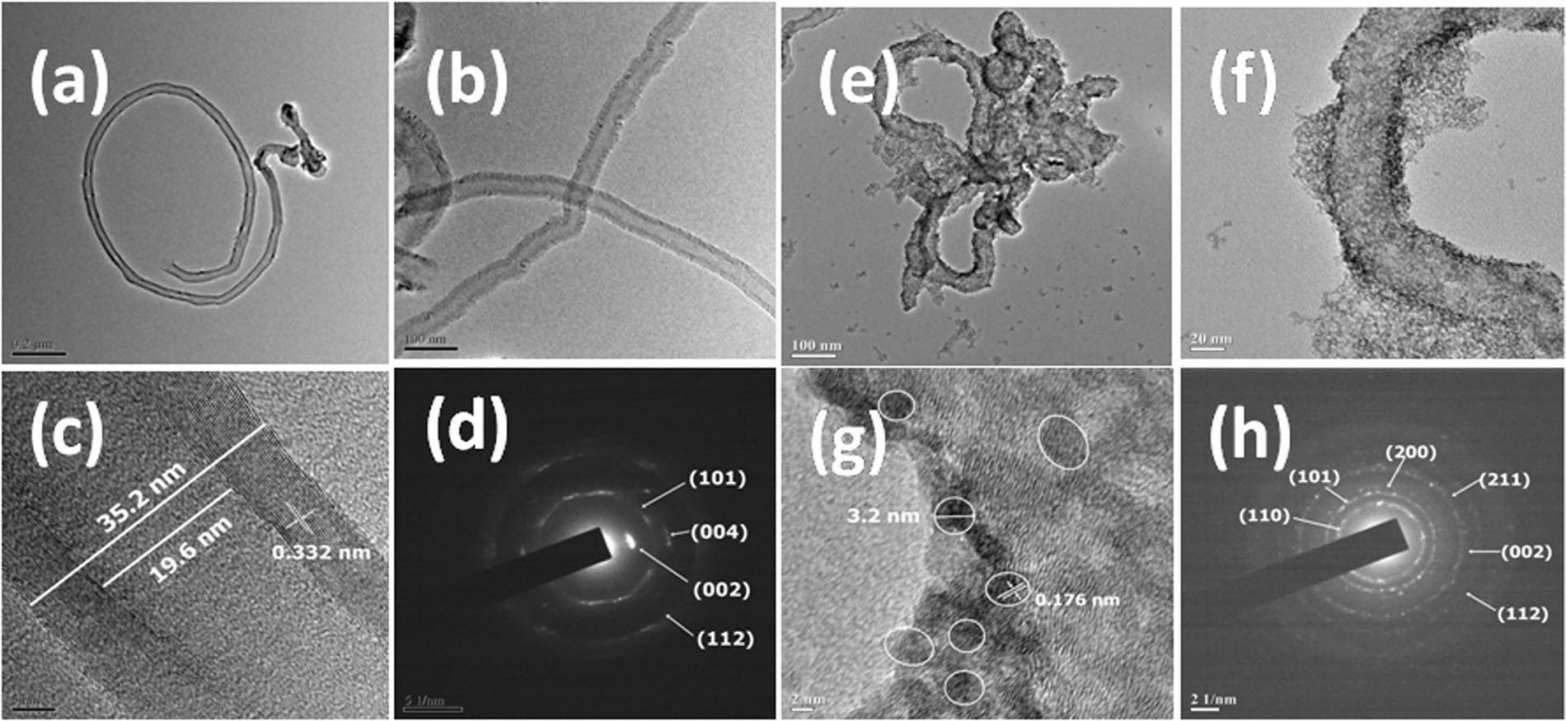
(**a**,**b**) TEM micrograph (**c**) HRTEM micrograph (**d**) SAED pattern of pristine CNT (**e**,**f**) TEM micrograph (**g**) HRTEM micrograph (**h**) SAED pattern of SnO_2_-CNT nanohybrid.

**Figure 3:**
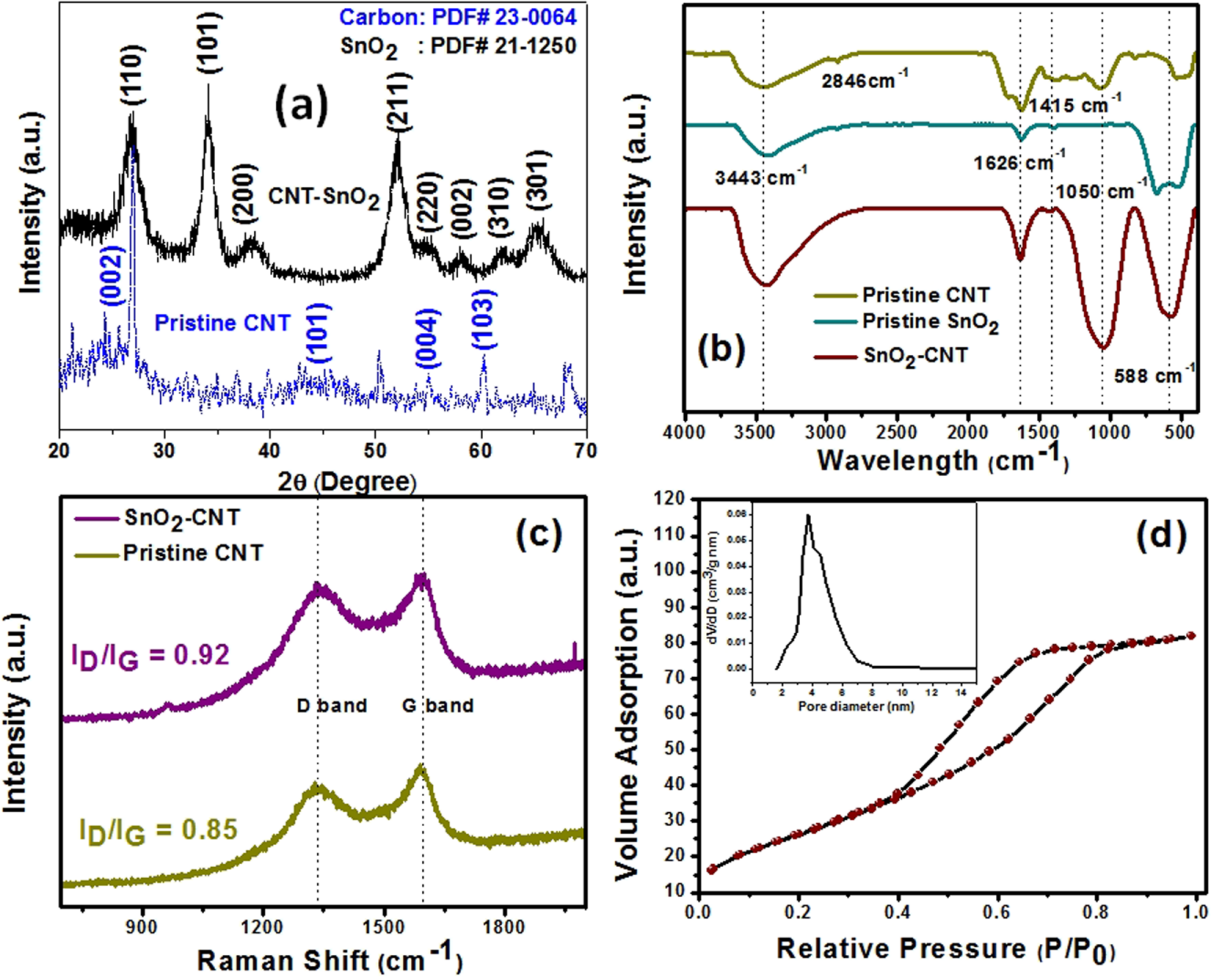
(**a**) XRD pattern of pristine CNT and SnO_2_-CNT nanohybrid (**b**)FT-IR spectra of CNT, SnO_2_ and SnO_2_-CNT nanocomposite (**c**) Raman spectra of pristine pristine CNT and SnO_2_-CNT (**d**) N_2_ adsorption-desorption isotherms at 77 K for SnO_2_ –CNT. Inset figure: particle size distributions derived from the desorption branch according to the BJH model.

The morphology of the as synthesized CNTs and SnO_2_-CNT nanocomposites were examined using transmission electron microscopy. The TEM micrographs (Fig. 2(a,b)) revealed quasi-aligned clean uniform >10 μm long hollow nanotubes formed due to CVD treatment of sunflower oil. These CNTs have an empty and uniform central core and open at one end. The nanotubes have long-range uniformity in the diameter with outer and inner diameters of ~35.2 nm and ~19.6 nm respectively. The HRTEM (Fig. 2(c)) suggested the as-grown nanotubes to be consisting of concentrically nested ~ 25 graphene sheets. The lattice fringes between two adjacent planes are 0.332 nm apart, which resembles the interlayer distance of (002) plane of the graphitic carbon. The SAED (Fig. 2(d)) revealed concentric rings that are characteristic of graphitic carbon (JCPDS 23-0064). Undesirable structures like amorphous carbon are negligible albeit with some structural defects. In SnO_2_-CNT, the tin oxide nanoparticles of nearly spherical shape are evenly distributed over the surface of CNTs (Fig. 2(e,f)**)**. The SnO_2_ nanoparticles are of uniform size with average particle size of 2.25 ± 0.3 nm (Fig. S4(a)). The lattice fringes showed a separation of 0.176 nm (Fig. 2(g)) which may be indexed to (211) plane of tetragonal rutile tin oxide (JCPDS 21-1250). The high crystallinity of SnO_2_-CNT nanocomposite was revealed by the SAED pattern (Fig. 2(h)) and the various lattice planes were identified and found to be in consistence with the XRD pattern **(**Fig. 3(a)**)**. The energy dispersive X-ray analysis (Fig. S4(b)) confirmed the composition of SnO_2_-CNT nanohybrid. The weight ratio of Sn and C was approximately 0.76 which indicated some loss of carbon may be due to heat treatment. Also the atomic ratio of O to Sn was found to be 1.26 indicating a high number of oxygen vacancies in the synthesized sample^43^. A comparative study of the morphologies of the SnO_2_-CNT nanohybrids with recent reports has been summarized in Table S1 (ESI). It was evident that low cost, biogenic, additive free fabrication of SnO_2_-CNT resulted better control over hybridization and growth of SnO_2_ nanoparticles, keeping the morphological characteristics of the CNT unscathed.

The X-ray diffraction pattern of the synthesized CNT and SnO_2_-CNT nanohybrid is shown in Fig. 3(a). Pristine CNT showed Bragg’s diffractions at 2θ values 26.52°, 44.61°, 54.67° and 59.7° corresponding to the (002), (101), (102) and (103) crystallographic planes of hexagonal graphitic carbon (JCPDS Card No 23-0064). The dominant diffraction planes of SnO_2_-CNT were indexed to be (110), (101), (200), (211), (220), (002), (310) and (301) of rutile tetragonal tin oxide (JCPDS Card No. 21-1250) possessing space group P4_2_/mnm (136), lattice parameters a = 4.738 Å and c = 3.188 Å. Comparing with the XRD pattern of pristine CNT, the distinct reflection peak of (002) facet of hexagonal graphitized carbon seemed to be overlapping with (110) facet of SnO_2_^44^. The decrease in FWHM values of the diffraction peaks after carbonization indicated the increase in crystallinity of the sample^45^. The average crystallite size is calculated by using the Debye-Scherrer’s equation, $D=\frac{k\lambda }{\beta cos\theta }$ where, D is the crystallite size in nanometers, k is the shape factor (0.89), λ is the wavelength of *CuK*α radiation (λ = 1.54056 Å), β is full width at half maximum (FWHM) of the particular peak and θ is the Bragg’s angle and was found to be 3.28 nm.

The FT-IR spectra of the as prepared compounds are shown in Fig. 3(b). The pristine CNT showed strong vibrations at 3443 cm^−1^, 1620–1695 cm^−1^ and 1050 cm^−1^ which may be attributed to the O-H, C=C and C-O stretching vibrations respectively. This signifies that the CNT surface may contain some surface functional groups like -OH, -COOH, -CHO *etc*. Low intensity peaks at 2846 cm^−1^ and 2914 cm^−1^ may correspond to the -CH and =CH vibrations and moderate intensity bands at 1358–1460 cm^−1^ are characteristics of CH_2_/CH_3_ banding vibrations. Pristine SnO_2_ showed a strong band at 588 cm^−1^ which is characteristic of Sn-O-Sn/Sn-OH stretching vibrations. Strong hydroxyl stretching (3443 cm^−1^) and bending (1626 cm^−1^) have appeared due to the physically absorbed H_2_O on SnO_2_ surface^17^. The appearance of the C-O band (1050 cm^−1^) retaining the characteristic Sn-O band (588 cm^−1^) in SnO_2_-CNT confirmed the composite formation.

Raman Spectroscopy is a sensitive and informative technique to investigate the interaction between carbon based materials with other components. The Raman spectra (Fig. 3(c)) of the pristine CNT and SnO_2_-CNT exhibited characteristic first order D- and G-bands at ~1333.39 cm^*−*1^ and ~1596.37 cm^*−*1^ respectively, indicating the carbon backbone. The G band corresponds to the tangential stretching (E_2g_) mode of the highly oriented pyrolytic graphite. The D-peak assigned to *A*_1g_ mode originates from the disorder in the sp^2^-hybridized carbon and indicated a lattice distortion in the graphitic carbon^46^. Therefore, the intensity ratios (I_D_/I_G_) are a measure of the extent of modification and the associated defects on the CNT. Notably, The I_D_/I_G_ ratio for pristine CNT and SnO_2_-CNT were found to be 0.85 and 0.92 respectively. The little increase in I_D_/I_G_ ratio implies a decrease of the average size of sp^2^ domains and increase in the atomic ordering of crystallinity of the CNT surface after being covered with SnO_2_^47^. Meanwhile, a slight up-shift of the G-band (From 1589–1603 cm^−1^) was also observed with SnO_2_ deposition which indicated efficient charge transfer between CNTs and SnO_2_^47^_._

The surface area and pore structures of the synthesized SnO_2_-CNT nanohybrids were investigated using BET surface area analysis **(**Fig. 3(d)**)**. The materials showed typical type IV isotherms with H2 hysteresis loops which are characteristics of mesoporous materials having narrow mouths or channel-like pores structures. The percolation process *i*.*e*., the blocking of narrow mouth pores due to condensation of adsorbate at the neck resulted in the steepness of the desorption branches of H2 hysteresis loops^48^. The BET surface area of pure SnO_2_-CNT was found to be 9.76 × 10^6^ cm^2^/g. Also, SnO_2_-CNT exhibited high BJH pore diameter and mesopore volume of 3.7 nm and 0.136 cm^3^/g respectively. The high surface area and superior mesoporous nature are likely to benefit the adsorption behavior and catalytic performance of the SnO_2_-CNT nanacomposite.

### Oxidative adsorption of As (III) on SnO_2_-CNT nanocomposite

**Figure 4:**
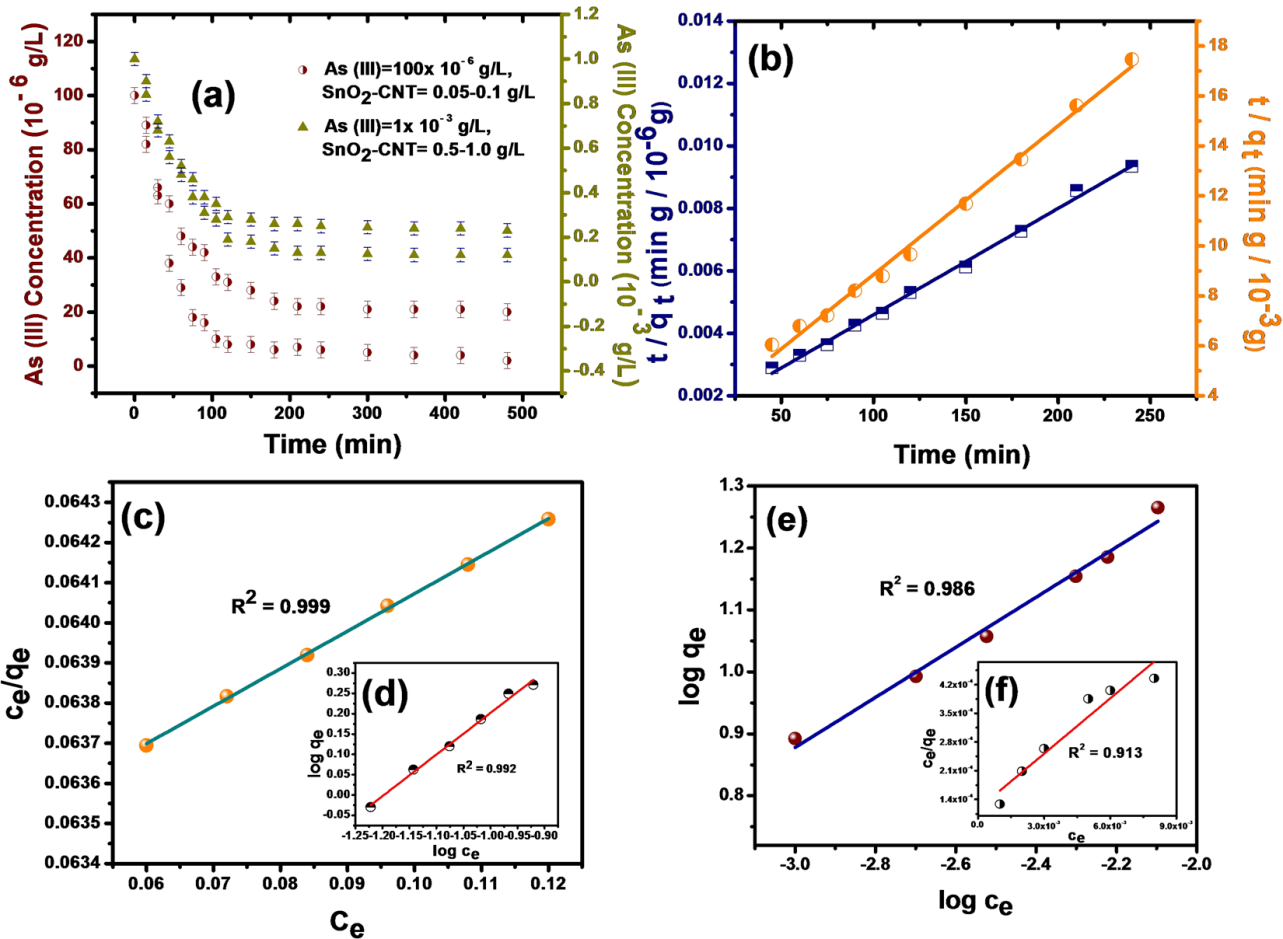
(**a**) Adsorption kinetics of arsenite on SnO_2_-CNT nanocomposite with different dosage and initial concentrations (**b**) The pseudo second order kinetic model fitting at C_0_ = 100 μg/L and C_0_ = 1 mg/L (**c**,**d**) Langmuir and Freundlich adsorption isotherms for C_0_ = 1 mg/L (**e**,**f**) Freundlich and Langmuir adsorption isotherms for C_0_ = 100 μg/L respectively.

The arsenic adsorption properties were investigated both at high arsenite concentrations as well as at low concentration ranges. Figure 4(a) demonstrated the decrease in arsenite concentration with time at two different initial concentrations. The As (III) removal efficiency was increased with increase in SnO_2_-CNT dosage and an optimal dose of 0.1 g/L could reduce the As (III) concentration from 100 μg/L to below WHO drinking water permissible limit within 120 min of treatment time. The SnO_2_-CNT adsorbent showed a good adsorption performance at high concentration range as well. A dose of 1.0 g/L could eliminate ~93% of the initial 1.0 mg/L As (III) concentration with in 120 min.

The pseudo first order and pseudo second order model were employed to study the adsorption kinetics of arsenite from the experimental data. The linear equations of the models are represented in Eq. (1) and Eq. (2) respectively.

1 $$\mathrm{Log}({{\rm{q}}}_{{\rm{e}}}-{{\rm{q}}}_{{\rm{t}}})=\,\log \,{{\rm{q}}}_{{\rm{e}}}-(\frac{{{\rm{k}}}_{1}}{2.303}){\rm{t}}$$

2 $$\frac{t}{{q}_{t}}=\frac{1}{{k}_{2}{q}_{e}^{2}}+(\frac{1}{qe})t$$

where q_e_ and q_t_ are the amounts of arsenic adsorbed per unit weight of adsorbent in equilibrium and at time t respectively. k_1_ and k_2_ are the rate constants of pseudo first order and pseudo second order kinetic model respectively.

The kinetic parameters obtained from the fitting of the experimental data are summarized in Table 1. From the correlation coefficients (R^2^) it was found that the pseudo second order model (Fig. 4(b)) fitted better than the pseudo first order model (Fig. S5(a,b)). Further, pseudo second order model showed a close agreement of the theoretical and the experimental values q_e_ for both the concentration ranges. The best fitting of the pseudo second order model suggested that the As (III) adsorption rate is dependent on both the concentrations of As species and the adsorption sites^49^. Moreover, chemisorption *i*.*e*., interaction through valency forces involving sharing or exchange of electrons between As (III) and SnO_2_-CNT may be responsible for the adsorption process^50^.

**Table 1:** Pseudo first order and pseudo second order kinetic model fitting parameters for As (III) adsorption.

Kinetic models	Kinetic parameters	Values for As(III) adsorption (C_0_ = 100 ppb)	Values for As(III) adsorption (C_0_ = 1 ppm)
Pseudo-first order	q_e_ experimental	19600 μg/g	25.20 mg/g
	q_e_ calculated	8511.38 μg/g	22.38 mg/g
	K_1_	9.21 × 10^−3^ min^−1^	2.17 × 10^−2^ min^−1^
	R^2^	0.795	0.980
Pseudo-second order	q_e_ calculated	20283 μg/g	24.45 mg/g
	K_2_	2.5 × 10^−6^ g μg^−1^ min^−1^	5.1 × 10^−4^ g mg^−1^ min^−1^
	R^2^	0.998	0.993

The adsorption capacities of the SnO_2_-CNT adsorbent in different concentrations of As (III) were evaluated using equilibrium adsorption isotherms. The adsorption data were fitted with the Langmuir and Freundlich isotherm models given by the Eqs (3) and (4) respectively.

3 $$\frac{{C}_{e}}{{q}_{e}}=\frac{1}{\,{q}_{m}{K}_{L}}+\frac{{C}_{e}}{{q}_{m}}$$

4 $$log\,{q}_{e}=\,\log \,{K}_{F}+\frac{1}{n}\,\log \,{C}_{e}$$

where C_e_ is the equilibrium concentration of As (III) in water sample and q_e_ is the equilibrium amount adsorbed. K_L_ and q_m_ are the Langmuir adsorption constant and maximum monolayer adsorption capacity respectively. K_F_ and n are Freundlich adsorption capacity and adsorption intensity respectively.

The experimental data were fitted with both Langmuir and Freundlich models and the parameters were presented in Table 2. It is evident from the R^2^ values that at the higher concentration range the adsorption data were fitted well with both Langmuir and Freundlich models (Fig. 4(c,d)). However, at low concentration range, Freundlich model (Fig. 4(e)) can describe the adsorption behavior effectively. The maximum adsorption capacity (q_m_) from the best fitted Langmuir model is found to be 106.95 mg/g. The separation factor (R_L_), given by, R_L_ = 1/(1 + K_L_ × C_0_) was found to be 0.87 (<1) implying favorability of the Langmuir adsorption process at higher concentration range. Also, 1/n is found to be 0.404 (<0.5) suggesting favorable Freundlich adsorption at low concentration range^51^. Thus among the various As (III) nanoadsorbents, SnO_2_-CNT demonstrated superior arsenite adsorption behavior both at low and high arsenic concentrations (Table S2 ESI).

**Table 2:** Langmuir and Freundlich parameters for As(III) adsorption over SnO_2_-CNT nanocomposite.

Isotherm models	parameters	Values for As(III) adsorption (Low concentration range)	Values for As(III) adsorption (High concentration range)
Langmuir model	q_m_ (mg/g)	22.22	106.95
	K_L_	358.6	0.148
	R_L_	0.025	0.87
	R^2^	0.913	0.999
Freundlich model	K_F_ (mg/g)	123.59	16.82
	1/n	0.404	1.024
	R^2^	0.986	0.992

The effect of change in pH and the presence of competing ions on the arsenite removal efficiency have also been scrutinized. Fig. S6(a) (ESI) showed the decrease in arsenic removal efficiency with increase in pH from 2 to 10. High adsorption capacity at low pH is because of the favorable protonation of the adsorbent surface, thereby, increasing the electrostatic attraction between the sorbate and sorbent^49^. However, the decrease in adsorption efficiency at high pH is may be due to the competing OH^−^ ions and hence high pH conditions are beneficial for desorption studies. Figure S6(b) (ESI) demonstrated the effect of co-existing anions on arsenic removal efficiency of the adsorbent. The concentrations of different interfering anions like Cl^−^, NO_3_^−^, SO_4_^2−^ and PO_4_^3−^ have been adjusted from 0.05 M to 0.2 M while the initial arsenite concentration (1.0 mg/L), adsorbent dose (1.0 g/L) and pH (7) were kept constant. Phosphate showed the maximum interference because of the chemical similarity with arsenite. However, the arsenic removal efficiency of the SnO_2_-CNT was found to be ~70% even in presence of 200 times higher PO_4_^3−^ concentration than that of As (III).

The regeneration and reusability of the adsorbent even after several adsorption-desorption cycles is an important criterion for practical applications. The regeneration was conducted using 0.1 M NaOH solution as eluent and even after five successive cycles, adsorption percentage was found to be almost constant (~86%) (Fig. S6(c) (ESI)). These results demonstrated the good regeneration potential of the adsorbent. Thus, SnO_2_-CNT possesses great adsorption efficiency even at very low concentration, good selectivity, easy regeneration and better reusability which render it as a superior adsorbent towards treating arsenic contaminated water in future.

### Plausible mechanism of oxidative adsorption

The nonionic nature of the As (III) species renders inefficiency towards adsorption technologies. Oxidative adsorption includes oxidation of As (III) to more favored As (V) species followed by simultaneous adsorption. The role of SnO_2_ in the photocatalytic adsorbent SnO_2_-CNT is to absorb sufficiently energetic light to produce photogenerated electron-hole pairs. The electron accepting capability of CNTs and the potential barrier across SnO_2_-CNT heterojunction further stabilizes the excitons. In aerated solutions, O_2_ serves as the primary electron acceptor to generate superoxide anion radicals (^.^O_2_^−^) which can actively take part in the oxidation process as shown below^52^.

$${{\rm{SnO}}}_{2}+{\rm{h}}{\rm{\nu }}\to {{\rm{SnO}}}_{2}({{\rm{e}}}_{{\rm{CB}}}^{-}+{{\rm{h}}}_{{\rm{VB}}}^{+})$$

$${{\rm{SnO}}}_{2}({{\rm{e}}}^{-})+{\rm{CNT}}\to {{\rm{SnO}}}_{2}+{\rm{CNT}}\,({{\rm{e}}}_{{\rm{\phi }}}^{-})$$

$${\rm{CNT}}\,({{\rm{e}}}_{{\rm{\phi }}}^{-})+{{\rm{O}}}_{2}\to {\rm{CNT}}+{{\rm{O}}}_{2}^{\cdot -}$$

$${{\rm{O}}}_{2}^{\cdot -}+{{\rm{H}}}_{2}{\rm{O}}\to {{\rm{HO}}}^{\cdot }+{{\rm{HO}}}_{2}^{\cdot }$$

$$2{{\rm{HO}}}_{2}^{\cdot }\to {{\rm{H}}}_{2}{{\rm{O}}}_{2}+{{\rm{O}}}_{2}$$

$${{\rm{H}}}_{2}{{\rm{O}}}_{2}\to 2{{\rm{HO}}}^{\cdot }$$

$${{\rm{H}}}_{3}{{\rm{AsO}}}_{3}+{{\rm{O}}}_{2}^{\cdot -}+2{{\rm{H}}}^{+}\to {{\rm{H}}}_{3}{{\rm{AsO}}}_{3}^{\cdot +}+{{\rm{H}}}_{2}{{\rm{O}}}_{2}$$

$${{\rm{H}}}_{3}{{\rm{AsO}}}_{3}+{{\rm{HO}}}^{\cdot }+{{\rm{H}}}^{+}\to {{\rm{H}}}_{3}{{\rm{AsO}}}_{3}^{\cdot +}+{{\rm{H}}}_{2}{\rm{O}}$$

$${{\rm{H}}}_{3}{{\rm{AsO}}}_{3}+{{\rm{SnO}}}_{2}({{\rm{h}}}_{{\rm{VB}}}^{+})\to {{\rm{H}}}_{3}{{\rm{AsO}}}_{3}^{\cdot +}$$

$${{\rm{H}}}_{3}{{\rm{AsO}}}_{3}^{\cdot +}+{{\rm{HO}}}^{\cdot }\to {{\rm{H}}}_{2}{{\rm{AsOO}}}_{4}^{-}+2{{\rm{H}}}^{+}$$

$${{\rm{H}}}_{3}{{\rm{AsO}}}_{3}^{\cdot +}+{{\rm{HO}}}^{\cdot }\to {{\rm{H}}}_{2}{{\rm{AsO}}}_{4}^{\cdot -}+2{{\rm{H}}}^{+}$$

$${{\rm{H}}}_{3}{{\rm{AsO}}}_{3}^{\cdot +}+{{\rm{O}}}_{2}+{{\rm{H}}}_{2}{\rm{O}}\to {{\rm{H}}}_{2}{{\rm{AsO}}}_{4}^{\cdot -}+{{\rm{O}}}_{2}^{\cdot -}+3{{\rm{H}}}^{+}$$

**Figure 5:**
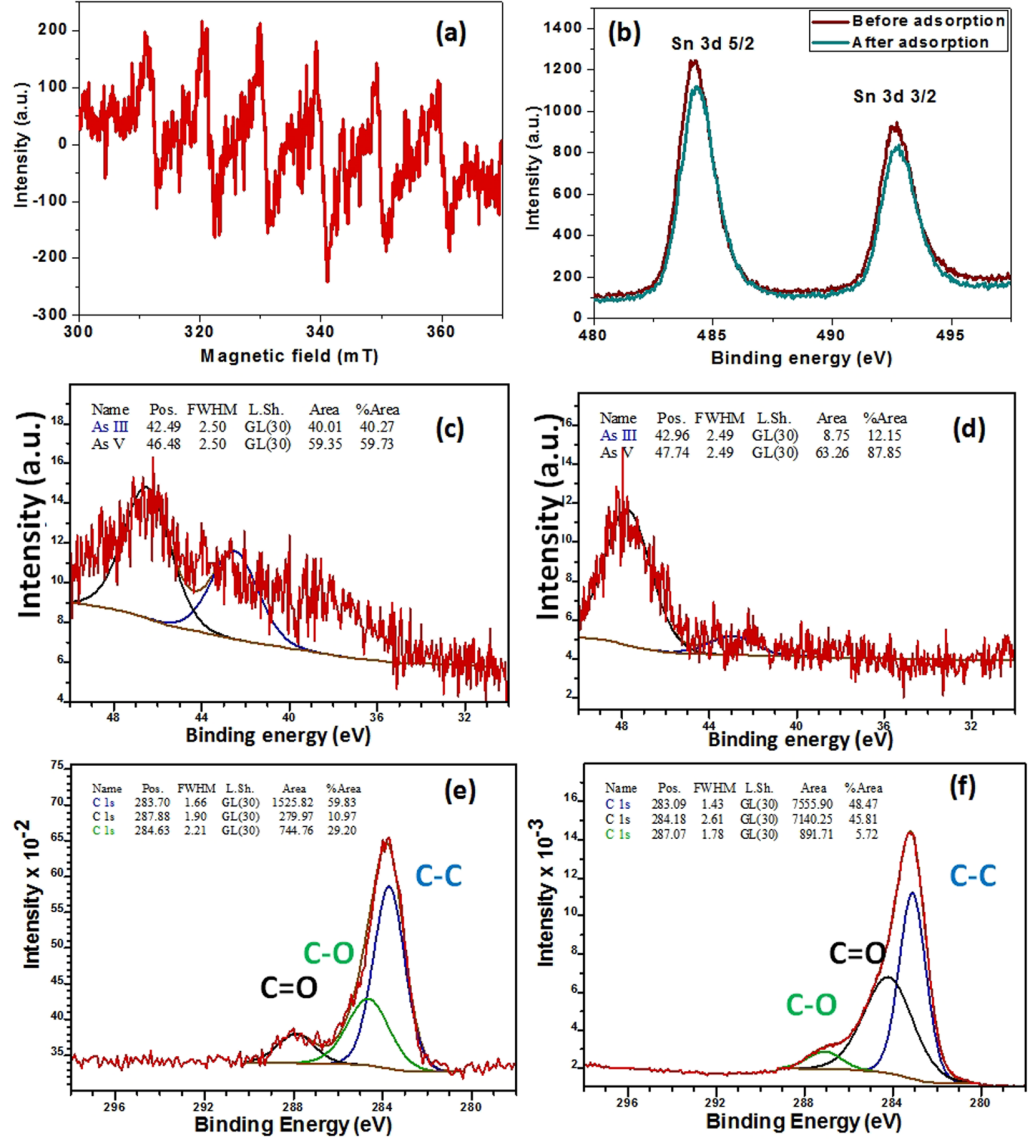
(**a**) PBN spin trapped ESR spectra for PBN-O_2_^·−^ under UV irradiation; High resolution XPS sprectra for (**b**) Sn 3d before and after adsorption (**c**) As 3d before adsorption (**d**) As 3d after adsorption (**e**) C 1s before adsorption and (**f**) C 1s after adsorption.

The identification of photo induced reactive species can be done from the ESR measurement using N-tert-butyl-α-phenylnitrone (PBN) as spin trapping agent. Figure 5(a) showed typical six peak ESR signal confirming the generation of superoxide anion radicals (^.^O_2_^−^) in the process. Further, X-ray photoelectron spectroscopy was utilized before and after the adsorption process to elucidate the adsorption mechanism. The binding energies of Sn 3d5/2 and Sn 3d3/2 were centered at ~484.2 eV and ~492.6 eV respectively which were assigned to Sn (IV) in SnO_2_ **(**Fig. 5(b)**)**. After adsorption, a slight decrease in the intensities of the Sn 3d spin-orbit doublet was observed along with a slight tilt towards high binding energy. This indicated the existence of strong chemical interaction between SnO_2_ and As species. The high resolution As 3d spectrum of the aqueous solution before and after the adsorption process was represented in Fig. 5(c,d) respectively. The signal can be deconvoluted into two individual component peaks centered at ~42.49 eV and ~46.48 eV assigned to As (III) and As (V) respectively. A momentous decrease in the intensity of the As (III) peak centered at ~42.96 eV after the adsorption was observed. Comparing the area under the curve before and after adsorption **(**Table S3 ESI**)**, it was evident that peak area of As (III) has been decreased from 40.27% to 12.15%, thereby, indicating oxidation of As (III) to As (V) during oxidative adsorption process. Further, The C 1 s spectrum before adsorption **(**Fig. 5(e)**)** can be deconvoluted into three peaks at ~283.7, ~284.6 and ~287.8 eV corresponding to three functional groups C-C, C-O and C=O respectively. The curve fitting parameters of C 1 s before and after adsorption **(**Table S3 ESI**)** demonstrated that C=O content decreases from 10.97% to 5.72% while C-O increases from 29.20% to 45.81% with arsenic adsorption, thereby, indicating chemical interaction during adsorption process. Thus, photocatalytic oxidation of As (III) over SnO_2_-CNT surface has been established and was found to be the driving force of the adsorption process. The neutral As (III) species (H_3_AsO_3_) has lesser affinity towards the adsorbent surface but the oxidation of As (III) to As (V) on SnO_2_-CNT surface generated anionic H_2_AsO_4_^−^ species. The hydroxyl groups present on adsorbent surface (as evident from FTIR spectra) may undergo ligand exchange with anionic H_2_AsO_4_^−^, thereby, facilitating enhanced adsorption behavior.

### Catalytic reduction of 4-nitrophenol

A popular line of investigation of catalytic performance of nanomaterials is catalytic reduction of nitroarenes. The reduction of 4-nitrophenol to 4-aminophenol by NaBH_4_ in presence of SnO_2_-CNT nanocatalyst have been monitored using UV-Vis spectroscopy. Aqueous solution of yellow colored 4-nitrophenol showed strong absorption at ~317 nm. In presence of NaBH_4,_ a red-shift was observed at ~400 nm due to the formation of nitrophenolate anion **(**Fig. S7(a)**)**^24^. In absence of catalyst, the reduction did not occur as the peak at 400 nm remained the same even after 2 days (Fig. S7(b)). However, in presence of catalyst, the peak at 400 nm diminished gradually with time along with the concomitant rise of characteristic peak of 4-aminophenol at ~300 nm. Hence, the changes in the absorbance at 400 nm have been screened to track the progress of the reduction reaction.

**Figure 6:**
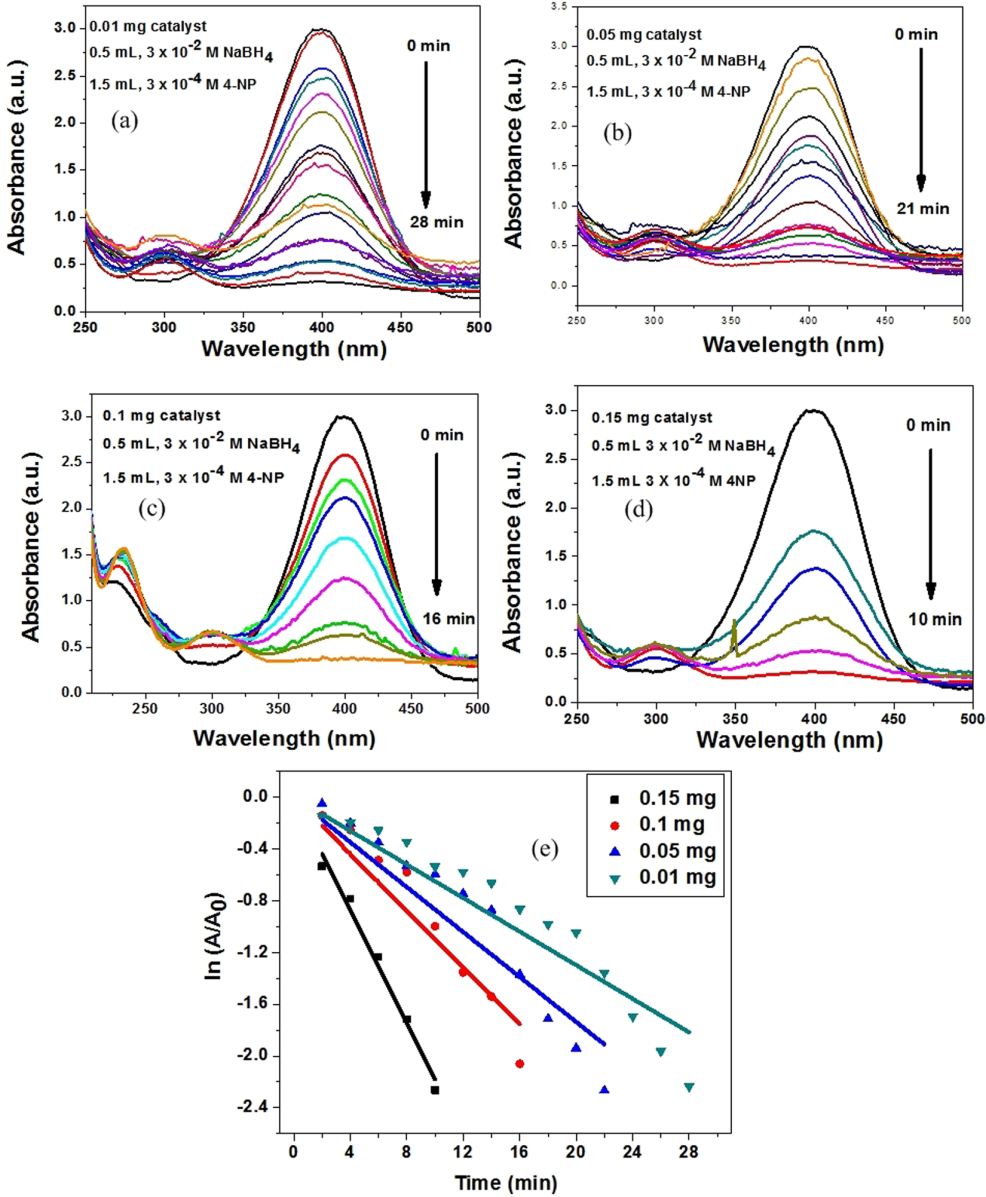
(**a**–**d**) UV-Vis absorption spectra for reduction of 4-nitrophenol reduction under varying catalyst doses (**e**) ln (A/A_0_) vs. time plot giving the comparative pseudo first order rate constants at different catalyst amounts.

The time-dependent reduction kinetics was studied with varied amounts of catalyst doses (Fig. 6(a–d)). The reaction conditions were optimized by varying concentrations of NaBH_4_ and 4-nitrophenol. The excess NaBH_4_ concentration (~100 times higher than that of 4-nitrophenol) induced the reaction to comply with pseudo-first order kinetics^26^. Since the ratio of concentration of 4-nitrophenol at time t (C) to that at time t = 0 (C_0_) is directly proportional to the corresponding absorbance ratio (A/A_0_), therefore, the pseudo first order kinetics can be estimated as follows

5 $${ln}(\frac{C}{{C}_{0}})=\,{ln}(\frac{A}{{A}_{0}})=-\,kt$$

where k is the pseudo first order rate constant and t is the reaction time in minutes. A_0_ is the initial absorbance of the nitrophenolate anion and A is that during the progress of reduction reaction. The nature of the ln (A/A_0_) vs. time plot was a straight line with negative slope **(**Fig. 6(e)**)** the rate constants at different catalyst dose (m) were calculated using Eq. (5). Moreover, the catalytic activity parameters (k_a_), the ratio of rate constants to catalyst doses (k/m), have been calculated for different catalyst doses and represented in Table 3. It has been found that the reduction rate became faster with an increase in catalyst amount, however, with a trivial decrease in catalytic activity. A comparative study of pseudo first order rate constants and activity parameter of the SnO_2_-CNT nanocatalyst with other reported catalysts has been summarized in Table S4 ESI. It is evident that the SnO_2_-CNT nanocatalyst showed better activity than most of the reported catalysts.

**Table 3:** Pseudo first order rate constants and catalytic activity parameters for the reduction of 4-nitrophenol at different catalyst amount.

Catalyst dose (mg)	Temperature (^0^C)	k (min^−1^)	k_a_ (min^−1^mg^−1^)	R^2^
0.01	25	0.077	7.7	0.93
0.05	25	0.109	2.18	0.95
0.1	25	0.159	1.59	0.97
0.15	25	0.219	1.46	0.99

**k** is the pseudo-first order rate constant, **k**_**a**_ is the catalytic activity parameter and **R**^**2**^ is the regression coefficient.

The reusability of the catalyst was tested by regenerating the catalyst after three successive rounds of catalytic reduction. Common regeneration method involving sonication, centrifugation, vacuum drying *etc*. were employed and the regenerated catalyst showed good catalytic activity with some loss at successive runs. The slight decrease in catalytic conversion after each run may be due to detachment of SnO_2_ nanopartcles from the CNT surface (Table S5 ESI). However, after three cycles of regeneration and reuse, appreciable catalytic activity was found which supports the potential practical applicability of the catalyst.

### Plausible mechanism of catalytic reduction

Previous reports explain the mechanism of the reduction on catalyst surface by Langmuir Hinshelwood model^53^. The substrates first get adsorbed over the catalyst surface, where the hydride transfer occurs followed by desorption of the product. In this respect, SnO_2_-CNT provided low crystallite size (3.28 nm), large surface area (9.76 × 10^6^ cm^2^/g) and surface functional groups which in turn directly affected the active site of the catalysis in reaction medium and hence increased the catalytic activity. Moreover, the reduced feasibility of the reduction process in absence of catalyst is due to the large potential barrier between the donor and the acceptor molecules. The heterojunction structure of the SnO_2_-CNT plays a crucial role for electronic interaction between the NaBH_4_ and 4-nitrophenol. The SnO_2_ can absorb electrons from BH_4_^**−**^ anions which may be transferred through CNT to the nitro groups of adsorbed 4-nitrophenol, thereby increasing the reduction rate. Hence, the small particle size, morphology, high surface area and the heterojunction structure of the SnO_2_-CNT are the factors responsible for the greater efficiency of the catalyst towards catalytic reduction of 4-nitrophenol.

### Photocatalytic degradation of Alizarin red S dye and Metronidazole

The applicability of the synthesized SnO_2_-CNT nanoheterojunctions to waste water treatment has also been explored by their degradation performance towards model water pollutants like Alizarin red S dye and metronidazole. The adsorption performance under dark condition for Alizarin red S and metronidazole were found to be only 16.5% and 11.8% respectively (60 min duration) and hence neglected in further studies. The photocatalytic performance was investigated under UV irradiation with initial contaminant concentrations in the range 10 mg/L to 80 mg/L and the catalyst dose in the range 0.1 g/L to 1.5 g/L. The progress of the degradation process was monitored by tracking the absorbance intensity at λ_max_ = 260 nm for Alizarin red S and λ_max_ = 322 nm for metronidazole^27,28^. The degradation efficiency has been determined using the following equation.

6 $$Degradation\,efficiency\,( \% )=(\frac{{A}_{0}-A}{{A}_{0}})\times 100$$

where A_0_ is the initial absorbance and A is absorbance at different interval of time.

**Figure 7:**
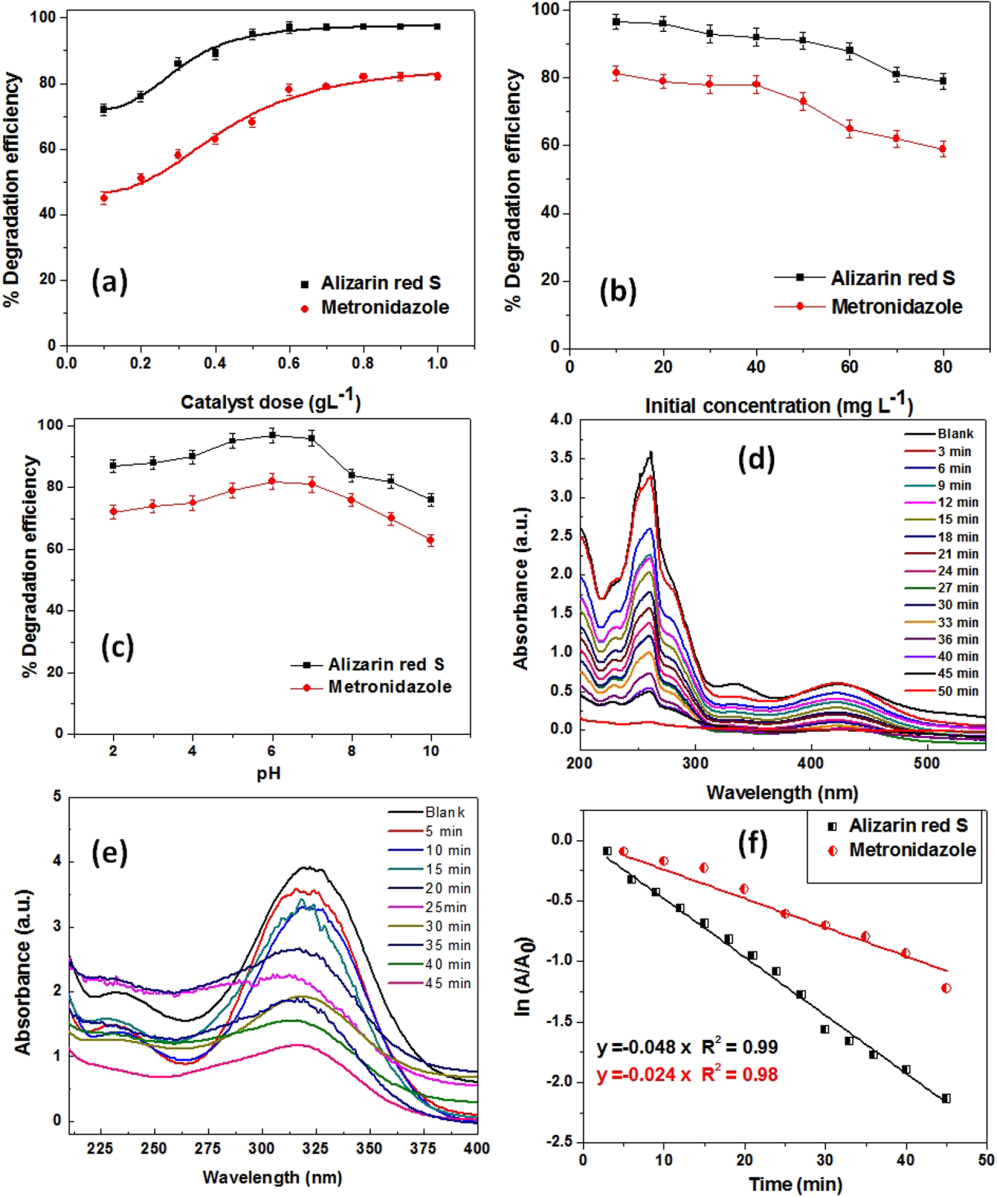
Effect of (**a**) Catalyst dose (**b**) initial contaminant concentration (**c**) initial pH on the photodegradation process (**d**) Absorption spectra during the degradation process of Alizarin red S (**e**) Absorption spectra during the degradation process of Metronidazole (**f**) Plot of ln (A/A_0_) vs. time for the photodegradation processes

The effect of catalyst amounts on the degradation efficiencies of Alizarin red S and metronidazole was evaluated by varying the catalyst concentration and keeping other parameters constant. Figure 7(a) showed steep increase in the percentage degradation for both Alizarin red S and metronidazole with increase in the amount of SnO_2_-CNT. A maximum degradation of ~97% for Alizarin red S and ~82% for metronidazole was achieved with catalyst amount 0.6 g/L and 0.8 g/L respectively. Whilst in the blank run, degradation efficiencies were found to be in the range 13–17% even after 60 min of illumination, suggesting the efficiency of the catalyst. The nearly stagnant degradation efficiencies beyond the optimized catalyst doses can be ascribed to the decreasing penetration power of the light with the increasing turbidity of the solution.

The effect of variation of initial contaminant concentrations on degradation efficiencies was studied by taking different concentrations of Alizarin red S and metronidazole in the range 10 mg/L to 80 mg/L with optimized catalyst doses (Fig. 7(b)). Maximum degradation efficiencies were recorded at initial concentration 50 mg/L for both the contaminants. The degradation performances were found to decrease with increase in initial contaminant concentrations. The reason being the increased number of adsorbed contaminants/degradation by-products on the surface of catalyst might have hindered the direct contact of reactive radicals (O_2_^.^/OH^.^) with the contaminant molecules, thereby, retarding the degradation^54^.

The effect of initial pH on photodegrdation of contaminants have also been investigated by varying the pH from 2–10. The pH of the solution was adjusted by appropriate amounts of 0.1 M HCl/NaOH solutions. Figure 7(c) demonstrated a slight increase in degradation efficiency for Alizarin red S while remained steady for Metronidazole upto pH 6. However, with further increase in pH, the degradation efficiencies were found to decrease steadily for both Alizarin red S and Metronidazole. The decrease in catalytic performance under high pH conditions may be attributed to the accumulation of negatively charged OH^−^ ions over the catalyst surface thereby repelling the electron rich contaminant molecules. Hence the optimization experiments result the best suited photodegradation conditions as, pH 6, 50 mg/L initial concentration and 0.6/0.8 g/L catalyst doses for Alizarin red S/Metronidazole respectively.

The absorbance intensities of Alizarin red S and Metronidazole as a function of time under optimized degradation conditions have been represented in Fig. 7(d,e). A gradual decrease of absorbance with increase in irradiation time can be noticed which is attributed to the decrease in concentration of the contaminants. The percentage degradation efficiencies calculated using the Eg.(6) was found to be ~97.2% and ~82% for Alizarin red S and Metronidazole in time 50 min and 45 min respectively. No new absorbance peaks have emerged during the degradation process, which indicated the mineralization of the degraded products. The extents of mineralization of both contaminants were examined by TOC analyzer during degradation process. A maximum TOC removal of 82.4% and 69.7% were found for Alizarin red S and Mentronidazole respectively (Table S6 ESI).

The kinetics of the photodegradation process catalyzed by SnO_2_-CNT has been investigated and a comparative plot of ln (A/A_0_) vs. time for Alizarin red S dye and Metronidazole has been shown in Fig. 7(f). The pseudo first order rate constants for Alizarin red S and Metronidazole were calculated using Eq. (5) and found to be 0.048 min^−1^ and 0.024 min^−1^ respectively. The photocatalytic activity parameters were also calculated using the equation k_a_ = k/C_catalyst_, and were found to be 0.08 Lmin^−1^g^−1^ and 0.03 Lmin^−1^g^−1^ for Alizarin red S and Metronidazole respectively.

It can be noted from Fig. S8 ESI, under UV irradiation conditions, the SnO_2_-CNT nanocomposite showed excellent degradation efficiencies for both Alizarin red S (~97.2%) and Metronidazole (~82%) while the pure SnO_2_ and CNT displayed poor performances even under prolonged UV irradiation (duration: 90 min). Moreover, the SnO_2_-CNT photocatalyst catalyst showed a decrease in degradation efficiencies (~78% and 63%) under direct solar irradiation condition (duration: 120 min). The catalyst showed good stability and maintained high activity over three consecutive cycles of regeneration and reuse. The degradation performance, however, decreased a bit with each run, which may be due to the dislodgement of the heterojunction.

To investigate the simultaneous multifunctional capability, the removal efficiencies of SnO_2_-CNT nanohybrids towards a mixture of contaminants like As (III), alizarin red S and Metronidazole have been examined. To a 50 mL solution of As (III) (1 mg/L), Alizarin Red S (50 mg/L) and Metronidazole (50 mg/L), approximately 1 g/L nanocomposite was added and maintaining the similar conditions as earlier, the removal efficiencies have been measured (Fig. S9 ESI). Due to competitive adsorption and substrate selectivity, a varying degree of removal efficiencies have been observed^55^. A sharp decrease in As (III) removal efficiency was evident due to competitive adsorption of organic contaminant molecules on the surface of the nanocomposite. This preferential adsorption may be attributed to the superior concentration and various polar functional groups of the organic molecules. Since the concentration of As (III) is very low as compared to the concentrations of Alizarin red S and metronidazole, their removal efficiencies were negligibly affected by the presence of As (III). Further, substrate selective activity was observed with SnO_2_-CNT nanocomposite, where removal of Alizarin red S being favored more over Metronidazole. The simultaneous removal efficiencies of As (III), Alizarin red S and Metronidazole were found to be ~62%, ~89% and 68%, respectively which are slightly lower than their individual counterparts (~93%, 97% and 82% respectively).

### Plausible mechanism of photodegradation

**Figure 8:**
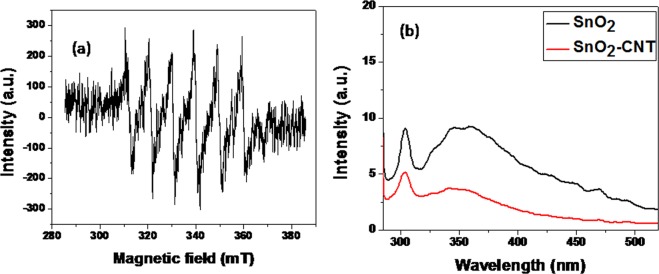
(**a**) PBN spin trapped ESR spectra for PBN-O_2_^·−^ under UV irradiation. (**b**) PL spectra of pristine SnO_2_ and SnO_2_-CNT at excitation wavelength of 272 nm.

In order to evaluate the mechanistic aspect of photodegradation process, the photo induced reactive species was identified using Electron Spin Resonance (ESR) spectroscopy. The active species formed during photodegradation process was trapped using N-tert-butyl-α-phenylnitrone (PBN) and ESR measurements were carried out to detect the generation of reactive radicals under UV irradiation (Fig. 8(a)). When a sample during an intermediate stage of degradation was exposed to UV irradiation, six typical ESR signals were detected indicating the generation of superoxide anion radical (^.^O_2_^−^)^56^.

Further, PL quenching effect was utilized to investigate the effect of heterojunction structure on recombination rate of photogenerated electron-hole pairs^56^. Commonly, high PL emission intensity refers to superior recombination of electron-hole pairs and hence lower photocatalytic performance. Figure 8(b) displayed the PL spectra of Pristine SnO_2_ NPs, and SnO_2_-CNT nanohybrids at an excitation wavelength of 272 nm. It was evident that both the samples exhibited similar shape of emission peaks at ~350 nm which corresponds to the band-gap recombination of electron-hole pairs in SnO_2_ generated under UV irradiation. The decrease in PL emission intensity indicated that the recombination rate of photogenerated excitons was much lower in SnO_2_-CNT than that in pristine SnO_2_. The decreased rate of recombination in SnO_2_-CNT would contribute to the enhancement of photocatalytic performance.

Based on the above discussion, the mechanism of photodegradation can be explicated by the electron transfer model in SnO_2_-CNT nanoheterojunctions under UV irradiation. The SnO_2_ nanoparticles can interact with the suitable frequency of the incident irradiation to undergo photoexcitation. Electrons from valence band (VB) of SnO_2_ may get transferred to the conduction band (CB), thereby, generating electron-hole pairs. However, the rapid recombination of the excitons, thus formed, lowers the activity of pristine SnO_2_. The electron accepting capacity and the charge mobility of the CNT makes the SnO_2_-CNT heterojunction crucial for superior synergistic performance. The relative positions of conduction band edge of SnO_2_ (−4.2 eV vs. vacuum) and the work function of CNT (5.6 eV) supports electron transfer from SnO_2_ (CB) to CNT^57^. The relocation of electrons from CB to the conjugated π-system of the CNT inhibits the charge recombination due to the potential barrier at the interface. The prolonged existence of the excitons, because of the electronic structure of the SnO_2_-CNT heterojunction, is responsible for greater photocatalytic performance. The electrons in the conduction band (e_**CB**_^**−**^) may reduce the surface oxygen (O_2_) to generate superoxide anion radicals (O_2_˙^−^) and the holes in the valence band (h _**VB**_^**+**^) may oxidize the water molecules (H_2_O) to generate OH˙ radicals. These highly active radicals can degrade the organic contaminants more effectively and in a very short time span. A schematic representation of the degradation process is represented as follows^54^.

$${{\rm{SnO}}}_{2}+{\rm{h}}{\rm{\nu }}\to {{\rm{SnO}}}_{2}({{\rm{e}}}_{{\rm{CB}}}^{-}+{{\rm{h}}}_{{\rm{VB}}}^{+})$$

$${{\rm{SnO}}}_{2}({{\rm{e}}}_{{\rm{CB}}}^{-})+{\rm{CNT}}\to {{\rm{SnO}}}_{2}+{\rm{CNT}}({{\rm{e}}}^{-})$$

$${\rm{CNT}}/{{\rm{SnO}}}_{2}({{\rm{e}}}^{-})+{{\rm{O}}}_{2}\to {\rm{CNT}}/{{\rm{SnO}}}_{2}+{{\rm{O}}}_{2}^{\cdot -}$$

$$2{{\rm{O}}}_{2}^{\cdot -}+4{{\rm{H}}}_{2}{\rm{O}}\to 2{{\rm{H}}}_{2}{{\rm{O}}}_{2}+2{{\rm{O}}}_{2}$$

$${{\rm{H}}}_{2}{{\rm{O}}}_{2}\to 2{{\rm{HO}}}^{.}$$

$${{\rm{SnO}}}_{2}({{\rm{h}}}_{{\rm{VB}}}^{+})+{{\rm{H}}}_{2}{\rm{O}}\to {{\rm{HO}}}^{\cdot }+{{\rm{SnO}}}_{2}$$

$${\rm{X}}+{{\rm{HO}}}^{\cdot }\to {{\rm{CO}}}_{2}+{{\rm{H}}}_{2}{\rm{O}}$$

$${\rm{X}}+{{\rm{e}}}_{{\rm{CB}}}^{-}\to {\rm{Reduced}}\,{\rm{product}}$$

$${\rm{X}}+{{\rm{h}}}_{{\rm{VB}}}^{+}\to {\rm{Oxidised}}\,{\rm{product}};\,{\rm{X}}={\rm{contaminants}}$$

### Identification of intermediate products of photodegradation

**Figure 9:**
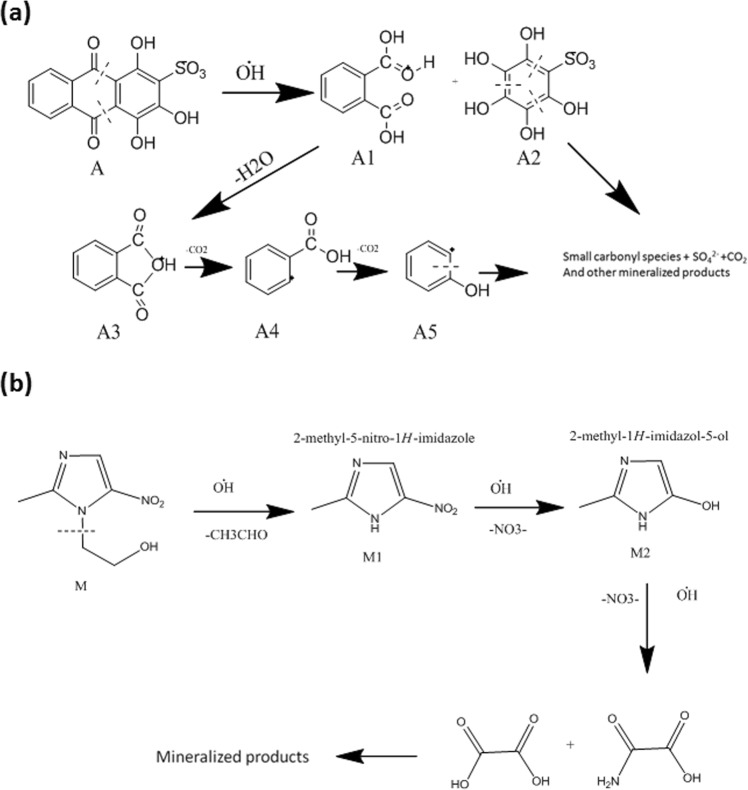
Plausible degradation pathways of (**a**) Alizarin red S and (**b**) Metronidazole.

To identify the intermediates generated during photodegradation process, samples of both Alizarin red S and Metronidazole were collected after 25 min and 20 min of illumination respectively. The samples were analyzed using LC-MS technique and the chromatrograms were furnished in Fig. S10 (ESI). The mass spectra of Alizarin red S taken at 11.1 min and 16.8 min of retention time have revealed major peaks at m/z–148.13, 104.08, 77.14 and m/z–122.08, 105.07, 94.06 and 78.02 corresponding to compound A3 and A4 respectively(Fig. S11(a,b)). From the mass spectral data, a plausible degradation pathway of Alizarin red S is represented in Fig. 9(a). The attack of photogenerated OH˙ radicals on the dye molecule have resulted the cleavage of C-C bond near C=O of Alizarin red S thereby forming phthalic acid (A1) and hydroxylated intermediate (A2)^58^. The polyhydroxylated intermediate (A2) being very unstable, immediately undergo cleavage to mineralization. The relatively stable phthalic acid undergo several steps of radical attack and elimination to form radical-ions of phthalic anhydride (A3), benzoic acid (A4), phenol (A5) and finally smaller carbonyls and mineralized products^59^.

The mass spectra of Metronidazole recorded at 4.2 min and 5.9 min of retention time revealed major peaks corresponding to compounds M1 (m/z 128.04) and M2 (m/z 98.03) (Fig. S11(c,d)). The LCMS alanysis, the probable fragmentation pathway of Metronidazole is represented in Fig. 9(b). The attack of hydroxyl radical on the parent molecule (M) have resulted the elimination of lateral N-ethanol group thereby forming 2-methyl-5-nitro-1H-imidazole (M1)^60^. Subsequent hydroxylation and denitration yielded 2-methyl-5-hydroxy-1H-imidazole (M2). Further degradation and ring opening of the imidazole moiety resulted in the formation of low molecular mass carboxylic acids which on decomposition gives mineralized products^61^. The m/z values of all the proposed intermediates correlates well with the observed LC-MS data and hence it is evidenced that the photodegradation products of both Alizarin red S and Metronidazole are low molar mass aliphatic carbonyls, carboxylic acids, amides *etc*. or mineralized products.

### Antimicrobial activity of SnO_2_-CNT Nanohybrids

In this study, SnO_2_-CNT nanohybrids exhibited synergistic activity against five bacterial and two fungal strains. The effect of antimicrobial susceptibility test was evaluated against standard antibiotics and summarized in Fig. S12(a,b).

**Figure 10:**
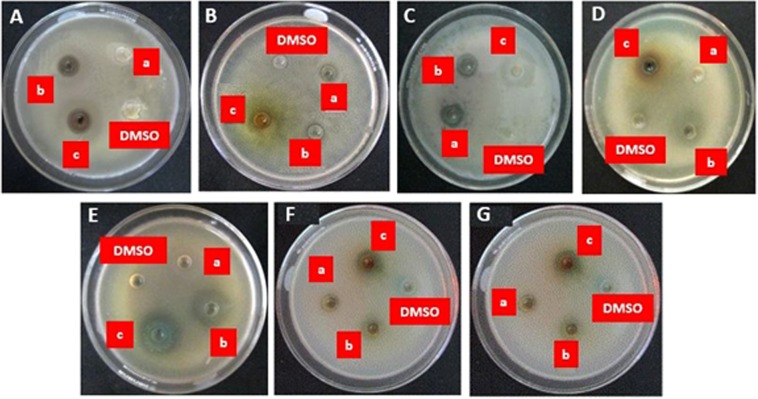
Antimicrobial activity of SnO_2_-MWCNT against (**A**) *Bacillus subtilis* (Bs s), (**B**) *Escherichia coli* (Ec c), (**C**) *Streptococcus pneumoniae* (St pn), (**D**) *Staphylococcus aureus* susp. *aureus* (St au), (**E**) *Pseudomonas aeruginosa* (Ps ae), (**F**) *Candida albicans* (C. albicans), and (**G**) *Aspergillus niger* (A. niger) where concentrations are (a) 0.01; (b) 0.03; (c) 0.04 M.

The biosynthesized SnO_2_-CNT nanocomposites exhibited a broad-spectrum activity with consistent microicidal efficiency against all the seven microbes, represented in Fig. S13(a,b) where 100% of the microbes were susceptible to the sample. Among them, the sample showed highest activity against *E*. *coli* with ZOI 32.23 ± 0.62 mm at 0.04 M **(**Fig. 10**)**. The bacterial strains were susceptible for *S*. *pneumoniae* at 0.01, 0.02 and 0.04 M concentrations with ZOI, 9.33 ± 0.88, 13.73 ± 0.93 and 17.47 ± 1.79 mm respectively. However, at 0.02 M lowest activities showed against *B*. *subtilis* with ZOI 10.17 ± 0.44 mm. In Fig. S13(b), antifungal activity of *C*. *albicans* displayed ZOI; 0, 13.40 ± 1.25, and 31.03 ± 0.78 mm at 0.01, 0.02 and 0.04 M respectively whereas the lab isolated fungus *A*. *niger* exhibited 0, 11.07 ± 1.35 and 31.03 ± 0.78 mm respectively. The results unveiled the potential activity of bio-fabricated SnO_2_-CNT nanohybrids towards clinically isolated pathogenic microorganisms. The plausible mechanism of microcidal activity might be the capturing of the microbial cells by quasi-aligned uniform >10 μm long nanotubes and disrupting the cell wall by SnO_2_ nanoparticles. Approximately 10 numbers of *E*. *coli* cells (each having diameter ≤1 μm) can be captured and wrapped tightly by a single CNT strand, thereby, inactivating the microorganism by rupturing the cell membranes^3^. The presence of SnO_2_ nanoparticles over the CNT surface may enhance the microcidal action by diffusing into the cell membrane and altering the cell functioning^62^. Moreover, the heterojuncion structure may lead to the generation of reactive oxygen species, which may interact with the cell wall, thereby resulting in lethal activity towards microbes^63^.

## Conclusion

In conclusion, we have synthesized SnO_2_-CNT nanoheterojunction using a biogenic, economically viable, environmentally sustainable strategy. Sunflower oil, a commonly available bio-precursor was utilized for the preparation of MWCNTs and were decorated with SnO_2_ nanoparticles using *Coccinia grandis* extracts. The various phytochemicals present in the plant extract played the role of complexing agents and no surfactants, capping agents, templates, solvents *etc*. were required to control growth of SnO_2_ nanoparticles over CNT surfaces.

The efficiency of bio-fabricated SnO_2_-CNT nanocomposites towards adsorption, catalysis and disinfection was exploited to provide one stop solution for the abatement water contaminants. The SnO_2_-CNT showed excellent efficiency of towards As (III) and a maximum Langmuir adsorption capacity of 106.95 mg/g was observed at high arsenite concentration (C_0_ = 1 mg/L). The nanoadsorbent was also found to be equally efficient in low arsenite concentration ranges (C_0_ = 100 μg/L) as it could bring down the arsenic concentration below maximum permissible limit within 120 minutes of operation time. It was found that the redox environment on SnO_2_-CNT surface is the active site for oxidation of As (III) to As (V) and the surface -OH groups of CNTs facilitated the adsorption process by anion exchange. One of the biggest challenges of application of CNTs in water treatment processes was their recollection after treatment. Hybridisation of CNTs with SnO_2_ nanoparticles has rendered the recovery of the adsorbent by gravitational sedimentation, coagulation and filtration after use. The SnO_2_-CNT nanoadsorbent showed effective removal in presence of interfering ions, good regeneration with NaOH and maintained a constant removal efficiency of ~86% after five consecutive adsorption-desorption cycles.

Moreover, the SnO_2_-CNT nano-heterojunctions have revealed their catalytic efficacy towards pollutants utilizing reduction of 4-nitrophenol as a model reaction. The manifestation of surface chemical properties of the heterojunction structure, and their ability to coerce the adsorbed nitrophenolates for a facile reaction has suggested the potential catalytic efficiency towards emerging organic water pollutants. Also, the catalyst showed great effectiveness towards photocatalytic degradation of model water contaminants like Alizarin red S dye and Metronidazole. Under UV irradiation, impressive degradation efficiencies of 97% and 82% were achieved for Alizarin red S and Metronidazole within 50 min of exposure. The catalyst retained good efficiency after three consecutive rounds of application, thereby, signifying potential applicability to treat waste water. Moreover, the degradation intermediates were identified using LC-MS technique and mechanism of degradation has been explained using ESR and PL studies.

Additionally, the nanocomposite displayed comparative antimicrobial action against both bacterial strains (*Bacillus subtilis*, *Escherichia coli*, *Streptococcus pneumonia etc*.), *and* fungal strains (*Candida albicans* and *Aspergillus niger*). These multifunctional proficiencies of SnO_2_-CNT nanohybrids suggest the promising applicability of hierarchical nano-heterojunctions to combat against natural, biological and man-made water contaminants.

## Supplementary information

Supplementary Information
