# Supplementary material for: Environmentally benign fabrication of SnO2-CNT nanohybrids and their multifunctional efficiency as an adsorbent, catalyst and antimicrobial agent for water decontamination
Source: Sci Rep. 2019 Sep 10;9:12935. doi: 10.1038/s41598-019-49181-2 (PMC6737164; doi:10.1038/s41598-019-49181-2)
Supplement: Supplementary file 1 — Supplementary Information [file 41598_2019_49181_MOESM1_ESM.docx]

**Electronic supplementary information**

Environmentally benign fabrication of SnO_2_-CNT nanohybrids and their multifunctional efficiency as an adsorbent, catalyst and antimicrobial agent for water decontamination

Md. Ahmaruzzaman^1^* Dipyaman Mohanta^1^ and Abhijit Nath^2^

^1^Department of Chemistry, National Institute of Technology, Silchar, Assam-788010, India

^2^Department of Chemistry, G.C. College, Silchar, Assam-788004, India

**Fig. S1**

**Fig. S1 FTIR spectrum of Coccinia *grandis* leaf extract**

**Fig. S2**

**Fig. S2. Plausible mechanism of formation of SnO_2_-CNT nanocomposite**

**Fig. S3**


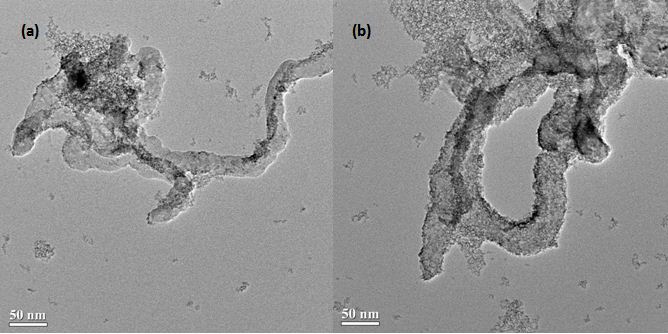


**Fig. S3 TEM micrographs of SnO_2_-CNT nanocomposite (a) before microwave treatment (b) after microwave treatment**

**Fig. S4**

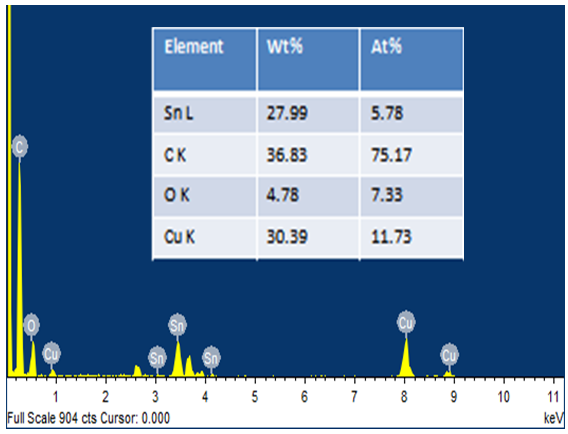


**(b)**

**(a)**

**Fig. S4 (a) Particle size distribution histogram (b) EDX spectrum of as synthesized SnO_2_-CNT**

**Fig.S5**

**(b)**

**(a)**

**Fig. S5 Pseudo first order kinetic plot for (a) initial arsenite concentration 100 μg/L (b) initial arsenite concentration 1 mg/L**

**Fig. S6**

**
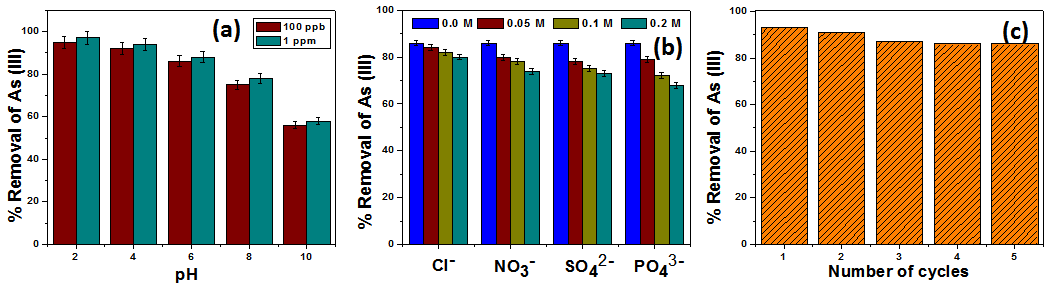
**

**Fig. S6 (a) Effect of pH on the adsorption efficiency at two different As (III) concentrations (b) Effect of interfering ions on adsorption efficiency at initial Arsenite concentration 1 mg/L. (c) Adsorption of As (III) after five successive cycles.**

**Fig. S7**

**Fig. S7 Absorbance spectra of 4-nitrophenol (a) in presence and absence of NaBH_4_ (b) In absence of catalyst**

**Fig.S8**

**Fig. S8 Comparative degradation efficiencies of Alizarin red S and Metronidazole under various catalysts, UV and solar irradiation conditions and recyclability test.**

**Fig.S9**

**Fig. S9 Comparative removal efficiencies of individual and mixed contaminants over SnO_2_-CNT nanocomposite**

**Fig. S10**

**
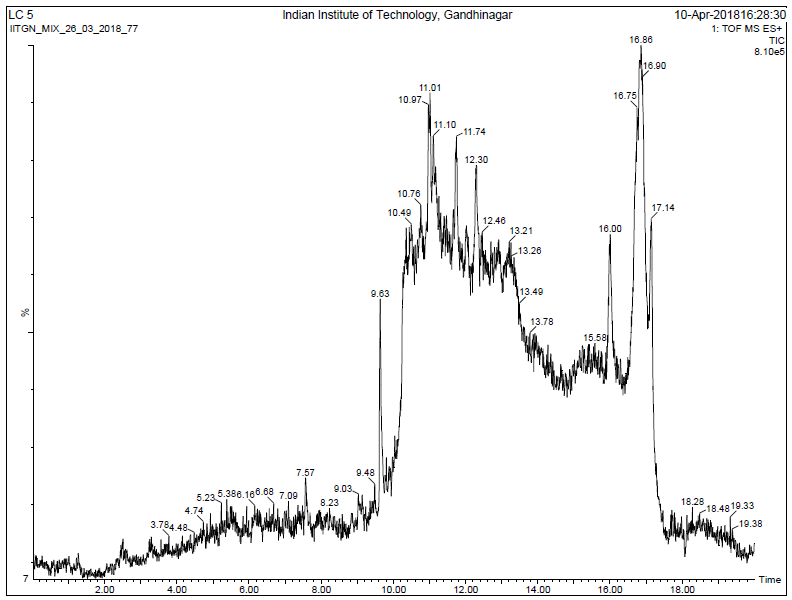

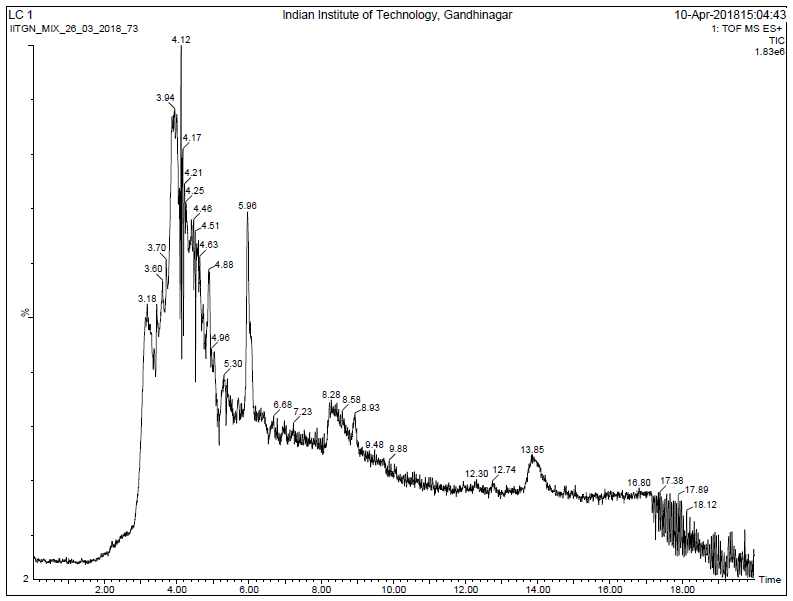
**

**(b)**

**(a)**

**Fig.S10 The LCMS chromatogram of photodegraded products of (a) Alizarin red S after 25 min of illumination (b) Metronidazole after 20 min of illumination.**

**Fig.S11**


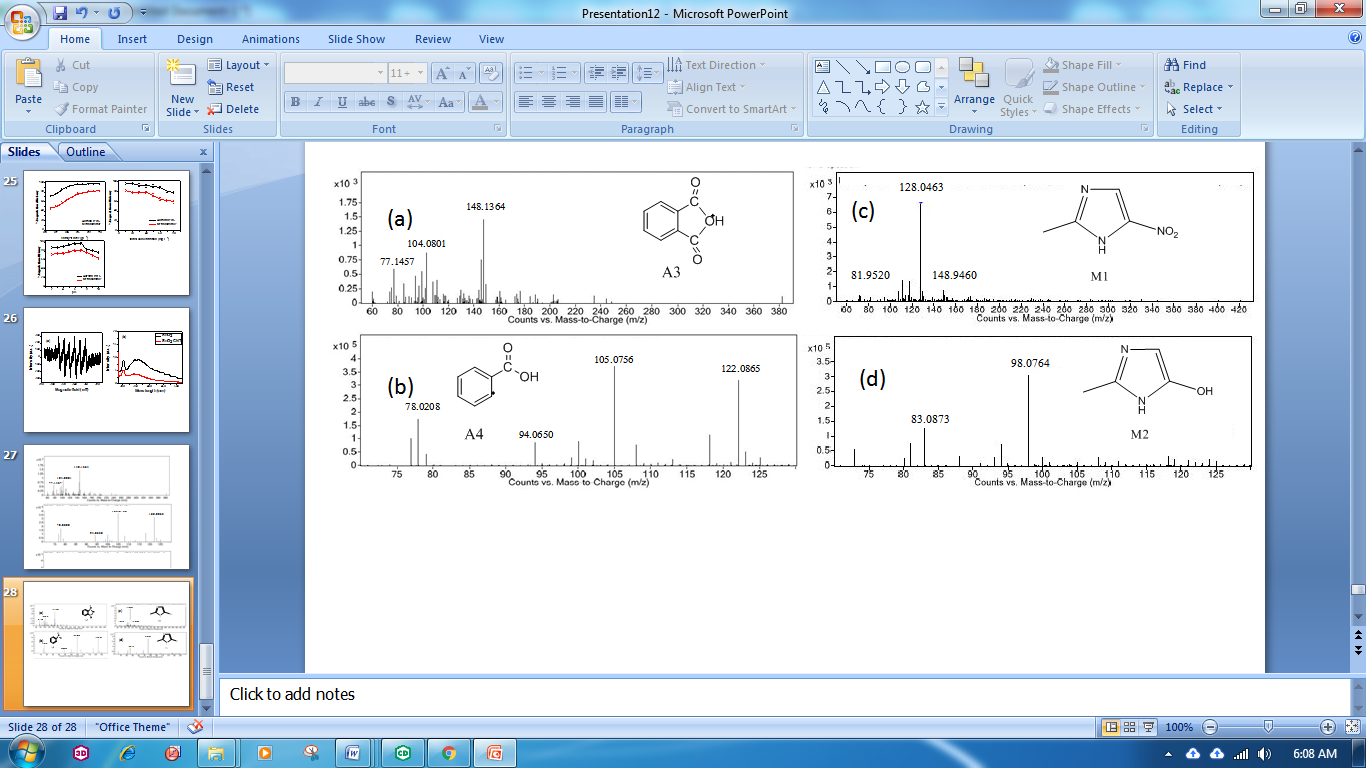


**Fig. S11 LC-MS spectra of different intermediates for degradation of (a, b) Alizarin red S (c, d) Metronidazole**

**Fig. S12**


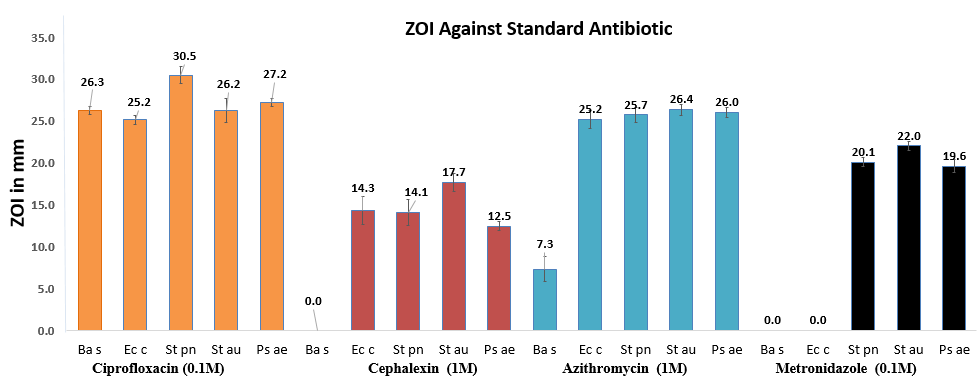


**Fig. S12(a). Effect of antibiotic susceptibility test on five pathogenic bacteria; *Bacillus subtilis* (Bs s), *Escherichia coli* (Ec c), *Streptococcus pneumoniae* (St pn)*, Staphylococcus aureus* susp. *aureus* (St au), *Pseudomonas aeruginosa* (Ps ae), and the values are with ± SE (n = 3)**


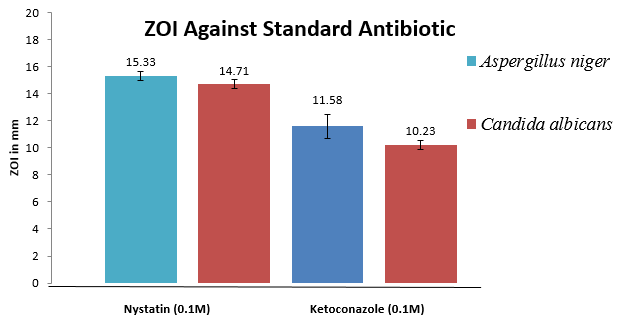


**Fig. S12(b) Effect of antibiotic susceptibility test of SnO_2_-MWCNT nanohybrids against two pathogenic fungi, *Aspergillus niger* and *Candida albicans***

**Fig. S13**


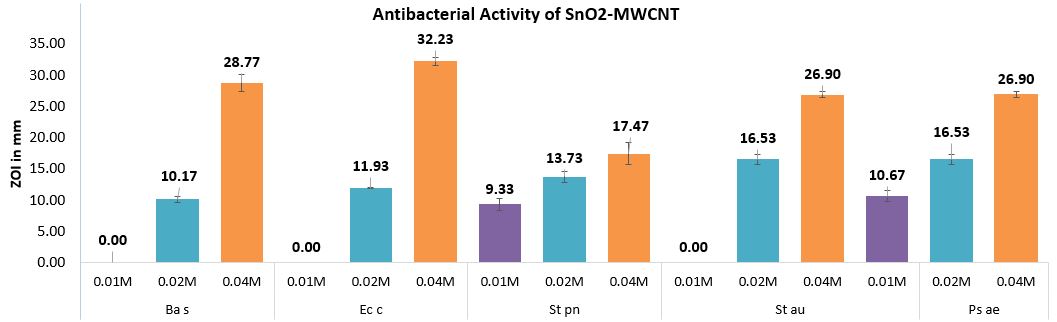


**Fig. S13(a) Effect of SnO_2_-MWCNT nanohybrids on growth of five pathogenic bacteria; *Bacillus subtilis* (Bs s), *Escherichia coli* (Ec c), *Streptococcus pneumoniae* (St pn)*, Staphylococcus aureus* susp. *aureus* (St au), *Pseudomonas aeruginosa* (Ps ae), and the values are with ± SE (n = 3)**


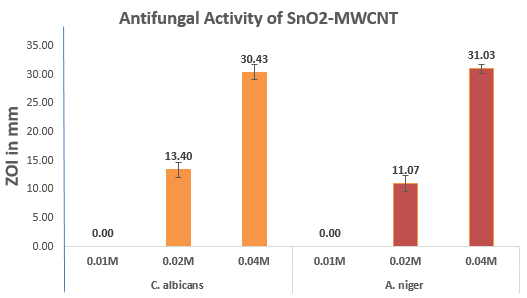


**Figure S13(b) Effect of SnO_2_-MWCNT nanohybrids on growth of two pathogenic fungi; *Candida albicans,* and *Aspergillus niger*, the values are with ± SE (n = 3)**

**List of Tables**

**Table S1. Comparison of morphological properties of SnO_2_-CNT nanostructures synthesized using different methods**

| Nanohybrids | Synthetic route | Pre-treatment /additives/reaction conditons | Dimensions/TEM micrograph  (Reprinted with permission from the respective publishing houses ) | References |
| --- | --- | --- | --- | --- |
| SnO_2_-CNT | CNT🡪 Purchased  SnO_2_-CNT🡪Sol-gel | HNO_3_(reflux)/0.7mL of 38% HCl in 40 mL of H_2_O/stirring | CNT🡪 20-40 nm in diameter SnO_2_🡪 2-4 nm diameter  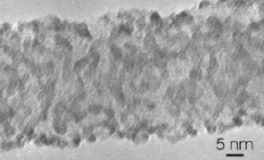 | Zhou, J. G., Fang, H. T., Maley, J. M., Ko, J. Y. P., Murphy, M., Chu, Y., ... & Sham, T. K. (2009). An X-ray absorption, photoemission, and Raman study of the interaction between SnO2 nanoparticle and carbon nanotube. *The Journal of Physical Chemistry C*, *113*(15), 6114-6117. |
|  |  |  |  |  |
| CNT@SnO_2_ core–sheath | CNT🡪 Purchased  SnO_2_-CNT🡪  Hydrothermal | HNO_3_ + H_2_SO_4_ solution (1:3) (reflux)/ 20 mg/mL CTAB solution/Autoclave(160^0^C for 24 h) | SnO_2_🡪 2-7 nm in diameter  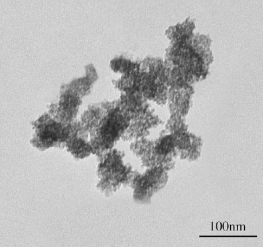 | Du, C., Chen, M., Cao, X., Yin, G., & Shi, P. (2009). A novel CNT@ SnO2 core–sheath nanocomposite as a stabilizing support for catalysts of proton exchange membrane fuel cells. *Electrochemistry Communications*, *11*(2), 496-498. |
|  |  |  |  |  |
| SnO_2_–CNT  Nano  composites | CNT🡪 Purchased  SnO_2_-CNT🡪Sol-gel | HNO_3_ + H_2_SO_4_ solution (1:3) (reflux)+NaSH in THF/ HNO_3_ + ethanol (1:6) (reflux)/stirring (RT, 24 h) | SnO_2_🡪 4 nm in diameter  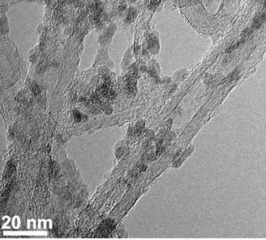 | Kim, S. P., Choi, M. Y., & Choi, H. C. (2015). Characterization and photocatalytic performance of SnO2–CNT nanocomposites. *Applied Surface Science*, *357*, 302-308. |
|  |  |  |  |  |
| CNT@SnO_2_ core-shell | CNT🡪 Purchased  SnO_2_-CNT🡪Sol-gel | HNO_3_(reflux )/ 0.7mL of 38% HCl in 40 mL of H_2_O/ Stirring (RT, 2 h) | SnO_2_🡪 2 nm in diameter  CNT🡪 40-60 nm in diameter, 1-10 μm in length  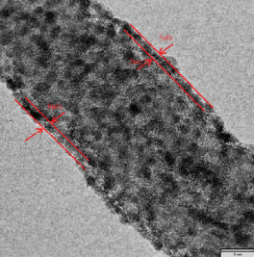 | Zhang, X., Zhu, H., Guo, Z., Wei, Y., & Wang, F. (2010). Design and preparation of CNT@ SnO2 core-shell composites with thin shell and its application for ethanol oxidation. *international journal of hydrogen energy*, *35*(17), 8841-8847. |
|  |  |  |  |  |
| CNT/SnO_2_ thick film | CNT🡪 Purchased  SnO_2_-CNT🡪  Snonochemical (4h) | Mechanical grinding/ calcintion (400^0^C, 2 h) | CNT🡪 10-30 nm in diameter, 10 nm in length  SnO_2_🡪 20-50 nm in diameter  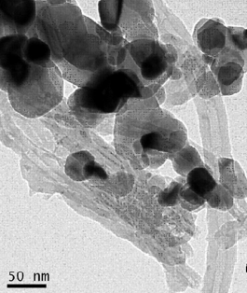 | Majumdar, S., Nag, P., & Devi, P. S. (2014). Enhanced performance of CNT/SnO2 thick film gas sensors towards hydrogen. *Materials Chemistry and Physics*, *147*(1-2), 79-85. |
|  |  |  |  |  |
| SnO_2_ nanocrystals anchored on MWCNT | CNT🡪 Purchased  SnO_2_-CNT🡪  Hydrothermal | H_2_SO_4_/HNO_3_ (3:1) (reflux)/ N_2_H_4_.H2O (3.84 g /500 mL)/Autoclave (150^0^C for 24 h) | SnO_2_🡪 3 nm in diameter  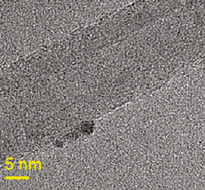 | Jin, Y. H., Min, K. M., Seo, S. D., Shim, H. W., & Kim, D. W. (2011). Enhanced Li storage capacity in 3 nm diameter SnO2 nanocrystals firmly anchored on multiwalled carbon nanotubes. *The Journal of Physical Chemistry C*, *115*(44), 22062-22067. |
|  |  |  |  |  |
| Mesoporous SnO_2_ on MWCNT | CNT🡪 catalytic decomposition of ethylene on Fe/Al_2_O_3_ catalyst  SnO_2_-CNT🡪  Hydrothermal | H_2_SO_4_/HNO_3_ (3:1) reflux/CTAB in alcohol-water system / Autoclave (160^0^C for 15 h) | SnO_2_ pore diameter 🡪3.5 nm  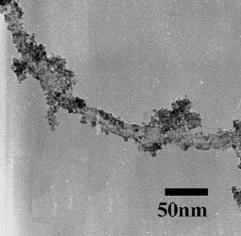 | Wen, Z., Wang, Q., Zhang, Q., & Li, J. (2007). In situ growth of mesoporous SnO2 on multiwalled carbon nanotubes: A novel composite with porous‐tube structure as anode for lithium batteries. *Advanced Functional Materials*, *17*(15), 2772-2778. |
|  |  |  |  |  |
| MWCNT filled and coated with SnO_2_ nanoparticles | CNT🡪 catalytic decomposition of methane  SnO_2_-CNT🡪Sol-gel | HNO_3_ reflux/ 0.7 mL of HCl(38%)/ stirring (1 h, 90^0^C) | SnO_2_🡪 3-5 nm in diameter  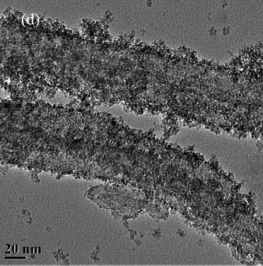 | Xu, C., Sun, J., & Gao, L. (2009). Synthesis of multiwalled carbon nanotubes that are both filled and coated by SnO2 nanoparticles and their high performance in lithium-ion batteries. *The Journal of Physical Chemistry C*, *113*(47), 20509-20513. |
|  |  |  |  |  |
| CNT/SnO_2_ core shell | CNT🡪 Catalytic decomposition of methane  CNT-SnO_2_🡪 Solvothermal | HNO_3_(reflux)/  1.25 mg/mL pyridine solution / Autoclave (140^0^C for 24 h) | CNT🡪 40-60 nm in diameter, 1-10 μm in length  SnO_2_🡪 10 nm in diameter  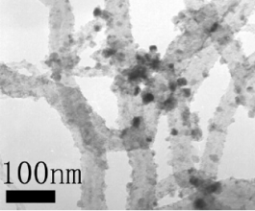 | Zhang, D., Pan, C., Shi, L., Mai, H., & Gao, X. (2009). Controllable synthesis and highly efficient electrocatalytic oxidation performance of SnO2/CNT core-shell structures. *Applied Surface Science*, *255*(9), 4907-4912. |
|  |  |  |  |  |
| SnO_2_ nanoparticles anchored on CNT | CNT🡪 Purchased  SnO_2_-CNT🡪Hydrothermal | Glucose as additive /Autoclave (160^0^C for 15 h)  / treatment in tubular furnace at 550^0^C for 3 h in Ar atmosphere | CNT🡪 20 nm in diameter,  SnO_2_🡪 2 nm in diameter  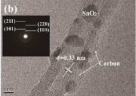 | Ma, C., Zhang, W., He, Y. S., Gong, Q., Che, H., & Ma, Z. F. (2016). Carbon coated SnO 2 nanoparticles anchored on CNT as a superior anode material for lithium-ion batteries. *Nanoscale*, *8*(7), 4121-4126. |
|  |  |  |  |  |
| Binder Free SnO2-CNT Composite | SnO_2_-CNT🡪  CVD of 10% mixture of CH_4_ in H_2_ | NiO in a 2 : 1  weight ratio dispersed in ethanol | CNT🡪 100-200 nm in diameter, more than 10 μm in length  SnO_2_🡪 5-10 nm in diameter  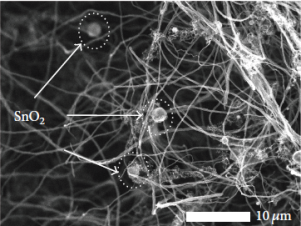 | Hernandez, D., Mendoza, F., Febus, E., Weiner, B. R., & Morell, G. (2014). Binder free SnO2-CNT composite as anode material for Li-Ion battery. *Journal of Nanotechnology*, *2014*. |
|  |  |  |  |  |
| SnO_2_@CNT  Composite | CNT🡪 Purchased  SnO_2_-CNT🡪Hydrothermal | Autoclave (150^0^C for 3 h)  / treatment in tubular furnace at 500^0^C for 2 h in Ar atmosphere | CNT🡪 20-30 nm in diameter,  SnO_2_🡪 5-7 nm in diameter  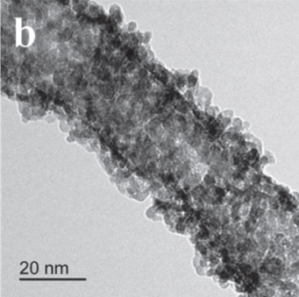 | Cheng, Y., Huang, J., Qi, H., Cao, L., Yang, J., Xi, Q., ... & Li, J. (2017). Adjusting the Chemical Bonding of SnO2@ CNT Composite for Enhanced Conversion Reaction Kinetics. *Small*, *13*(31). |
|  |  |  |  |  |
| SNO_2_/CNT hybrids | CNT🡪 Purchased  SnO_2_-CNT🡪Microwave | Acid treatment (HNO_3_, 120^0^C % min in microwave reactor)/ HCl, microwave 60^0^C, 5 min | CNT🡪 10-30 nm in diameter, more than 10 μm in length  SnO_2_🡪 3-5 nm in diameter  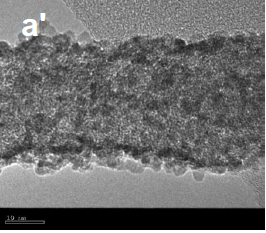 | Motshekga, S. C., Kesavan Pillai, S., Ray, S. S., Jalama, K., & Krause, R. W. (2011). An easy two-step microwave assisted synthesis of SnO2/CNT hybrids. John Wiley & Sons. |
|  |  |  |  |  |
| Pd/SnO_2_/CNT nanocomposites | CNT🡪 Purchased  SnO_2_-CNT🡪sol-gel | Acid treatment (HNO_3_, 80^0^C reflux, 12h/ Citric acid as additive/ calcinations at 500^0^C for 4h | CNT🡪 10-20 nm in diameter  SnO_2_🡪 8-20 nm in diameter  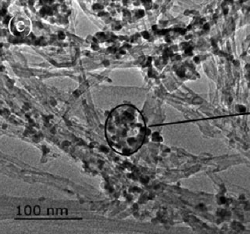 | Hu, Q., Liu, S., & Lian, Y. (2014). Sensors for carbon monoxide based on Pd/SnO2/CNT nanocomposites. *physica status solidi (a)*, *211*(12), 2729-2734. |
|  |  |  |  |  |
| SnO_2_-CNT nanohybrid | CNT🡪Chemical vapour deposition of sunflower oil (700^0^C) argon atmosphere  CNT-SnO_2_🡪 Sol-gel | No pretreatment/ *Coccinia grandis* leaf extract/ stirring (2 h, 70^0^C) | CNT🡪 20-35 nm in diameter, 10 μm in length  SnO_2_🡪 2.25 nm in diameter  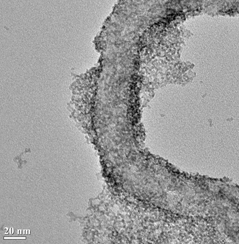 | This work |

**Table S2. Comparison of arsenic adsorption parameters of various reported adsorbents**

| Adsorbent | pH | Concentration | Surface area (m^2^g^-1^) | Temperature  (^0^C) | Model used to calculate adsorption capacity | Capacity  (mg/g) | Reference |
| --- | --- | --- | --- | --- | --- | --- | --- |
| CeO_2_-CNTs | 3.1 | 1000 ppm | 189 | - | Freundlich | 0.025 | Peng, X., Luan, Z., Ding, J., Di, Z., Li, Y., & Tian, B. (2005). Ceria nanoparticles supported on carbon nanotubes for the removal of arsenate from water. *Materials letters*, *59*(4), 399-403. |
| FeMnOx/RGO | 7 | 7 ppm | 548 | 25 ^0^C | Langmuir | 22.17 | Zhu, J., Lou, Z., Liu, Y., Fu, R., Baig, S. A., & Xu, X. (2015). Adsorption behavior and removal mechanism of arsenic on graphene modified by iron–manganese binary oxide (FeMnO x/RGO) from aqueous solutions. *RSC Advances*, *5*(83), 67951-67961. |
| Iron oxide-MWCNT composite | 7 | 1 ppm | -- | RT | Langmuir | 24.05 | Chen, B., Zhu, Z., Ma, J., Yang, M., Hong, J., Hu, X., ... & Chen, J. (2014). One-pot, solid-phase synthesis of magnetic multiwalled carbon nanotube/iron oxide composites and their application in arsenic removal. *Journal of colloid and interface science*, *434*, 9-17. |
| Iron Oxide- ORMOSIL - rGO | 7 | 2-4 ppm | 165.22 | RT | Langmuir | 38 | Sahu, T. K., Arora, S., Banik, A., Iyer, P. K., & Qureshi, M. (2017). Efficient and rapid removal of environmental malignant Arsenic (III) and industrial dyes using re-usable, recoverable ternary Iron Oxide–ORMOSIL-Graphene Oxide composite. *ACS Sustainable Chemistry & Engineering*, 5, 5912-5921. |
| Ceria-GO composite |  | 200 ppm | 129.42 | RT | Langmuir | 185 | Sakthivel, T. S., Das, S., Pratt, C. J., & Seal, S. (2017). One-pot synthesis of a ceria–graphene oxide composite for the efficient removal of arsenic species. *Nanoscale*, *9*(10), 3367-3374. |
| SnO_2_ nanospheres | 7 | 85.7 ppb – 1.32 ppm | 169.53 | RT | Langmuir | 112.7 | Zhang, G., Sun, W., Liu, L., Yang, W., Xu, Z., Li, Q., & Shang, J. K. (2015). Synthesis of tin oxide nanospheres under ambient conditions and their strong adsorption of As (iii) from water. *Dalton Transactions*, *44*(41), 18207-18214. |
| SnO_2_- CNT | 7 | 100 ppb- 1 ppm | 976 | RT | Langmuir | 106.95 | This work |

**Table S3 XPS fitting parameters for Sn 3d, As 3d and C 1s before and after arsenite adsorption.**

| Valence state | Assignment | Binding energy (eV) | FWHM | Relative content (%) |
| --- | --- | --- | --- | --- |
| Sn 3d (Before adsorption) | 3d 5/2 | 484.2 | 1.40 | 59.27 |
|  | 3d 3/2 | 492.6 | 1.40 | 40.73 |
| Sn 3d (After adsorption) | 3d 5/2 | 484.4 | 1.52 | 60.11 |
|  | 3d 3/2 | 492.8 | 1.48 | 39.89 |
| As 3d (Before adsorption) | As (V) | 46.48 | 2.50 | 59.73 |
|  | As (III) | 42.49 | 2.50 | **40.27** |
| As 3d (After adsorption) | As (V) | 47.74 | 2.49 | 87.85 |
|  | As (III) | 42.96 | 2.49 | **12.12** |
| C 1s (Before adsorption) | C-C | 283.70 | 1.66 | 59.83 |
|  | C-O | 284.63 | 2.21 | 29.20 |
|  | C=O | 287.88 | 1.90 | 10.97 |
| C 1s (After adsorption) | C-C | 283.09 | 1.43 | 48.47 |
|  | C-O | 284.18 | 2.61 | 45.81 |
|  | C=O | 287.07 | 1.78 | 5.72 |

**Table S4. Comparative study of pseudo first order rate constants and activity parameter of the SnO2-CNT nanocatalyst with other reported catalysts**

| Catalyst | Rate constant (s^-1^) | Activity parameter (g^-1^s^-1^) | References |
| --- | --- | --- | --- |
| Cu_2_O-Cu-CuO nanocomposite | 10.4 x 10^-3^ | 20.7 | Sasmal, A. K., Dutta, S., & Pal, T. (2016). A ternary Cu 2 O–Cu–CuO nanocomposite: a catalyst with intriguing activity. *Dalton Transactions*, *45*(7), 3139-3150. |
| Au-Fe_3_O_4_ | 10.1 x 10^-3^ | 11.22 | Lin, F. H., & Doong, R. A. (2011). Bifunctional Au− Fe3O4 heterostructures for magnetically recyclable catalysis of nitrophenol reduction. *The Journal of Physical Chemistry C*, *115*(14), 6591-6598. |
| Pd-rGO | 4.5 x 10 ^-3^ | 15.0 | Bramhaiah, K., & John, N. S. (2013). Hybrid films of reduced graphene oxide with noble metal nanoparticles generated at a liquid/liquid interface for applications in catalysis. *RSC Advances*, *3*(21), 7765-7773. |
| Oh Cu_2_O | 1.23 x 10^-2^ | 123.6 | Aditya, T., Jana, J., Singh, N. K., Pal, A., & Pal, T. (2017). Remarkable Facet Selective Reduction of 4-Nitrophenol by Morphologically Tailored (111) Faceted Cu2O Nanocatalyst. *ACS Omega*, *2*(5), 1968-1984. |
| SnO_2_-CNT | 1.28 x 10^-2^ | 128.33 | This work |

**Table S 5 Regeneration potential of the SnO2-CNT nanocatalyst after consecutive runs of catalytic cycles**

| Sl. No. | Time | Rate constant (min^-1^) | Percentage reduction |
| --- | --- | --- | --- |
| 1 | 10 | 15.9 x 10^-2^ | 99.2 |
| 2 | 10 | 11.3 x 10^-2^ | 96.1 |
| 3 | 11 | 8.6 x 10^-2^ | 92.3 |

**Table S6 Total organic carbon content of Alizaren red S and Metronidazole before and after degradation**

| Contaminants | Total carbon before degradation (ppm) | Total carbon after degradation (ppm) | TOC removal (%) |
| --- | --- | --- | --- |
| Alizarin red S | 30.58 | 5.39 | 82.4 |
|  |  |  |  |
| Metronidazole | 26.73 | 8.1 | 69.7 |
